# Controlling the expression of heterologous genes in *Bdellovibrio bacteriovorus* using synthetic biology strategies

**DOI:** 10.1111/1751-7915.14517

**Published:** 2024-06-27

**Authors:** Sergio Salgado, Natalia Hernández‐Herreros, M. Auxiliadora Prieto

**Affiliations:** ^1^ Interdisciplinary Platform for Sustainable Plastics towards a Circular Economy‐Spanish National Research Council (SusPlast‐CSIC) Madrid Spain; ^2^ Department of Microbial and Plant Biotechnology Polymer Biotechnology Group, Margarita Salas Center for Biological Research (CIB‐CSIC) Madrid Spain

## Abstract

*Bdellovibrio bacteriovoru*s HD100 is an obligate predatory bacterium that preys upon Gram‐negative bacteria. It has been proposed to be applied as a “living antibiotic” in several fields such as agriculture or even medicine, since it is able to prey upon bacterial pathogens. Its interesting lifestyle makes this bacterium very attractive as a microbial chassis for co‐culture systems including two partners. A limitation to this goal is the scarcity of suitable synthetic biology tools for predator domestication. To fill this gap, we have firstly adapted the hierarchical assembly cloning technique Golden Standard (GS) to make it compatible with *B. bacteriovorus* HD100. The chromosomal integration of the Tn*7* transposon's mobile element, in conjunction with the application of the GS technique, has allowed the systematic characterization of a repertoire of constitutive and inducible promoters, facilitating the control of the expression of heterologous genes in this bacterium. PJ_ExD_/EliR proved to be an exceptional promoter/regulator system in *B. bacteriovorus* HD100 when precise regulation is essential, while the synthetic promoter P_BG37_ showed a constitutive high expression. These genetic tools represent a step forward in the conversion of *B. bacteriovorus* into an amenable strain for microbial biotechnology approaches.

## Graphical abstract

The predatory bacteria *B. bacteriovorus* HD100 shows potential for biotechnological applications. We adapted the Golden Standard hierarchical assembly cloning technique for *B. bacteriovorus HD100*HD100 by incorporating the Tn7 transposon's mobile element into its chromosome. Our engineered strains were thoroughly assessed using flow cytometry, confirming their strong predatory efficacy and holding significant promise for new applications.

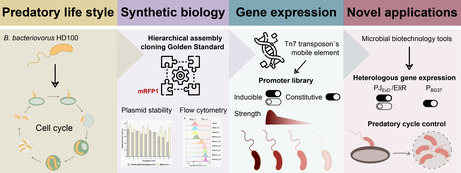

## INTRODUCTION

**FIGURE 1:**
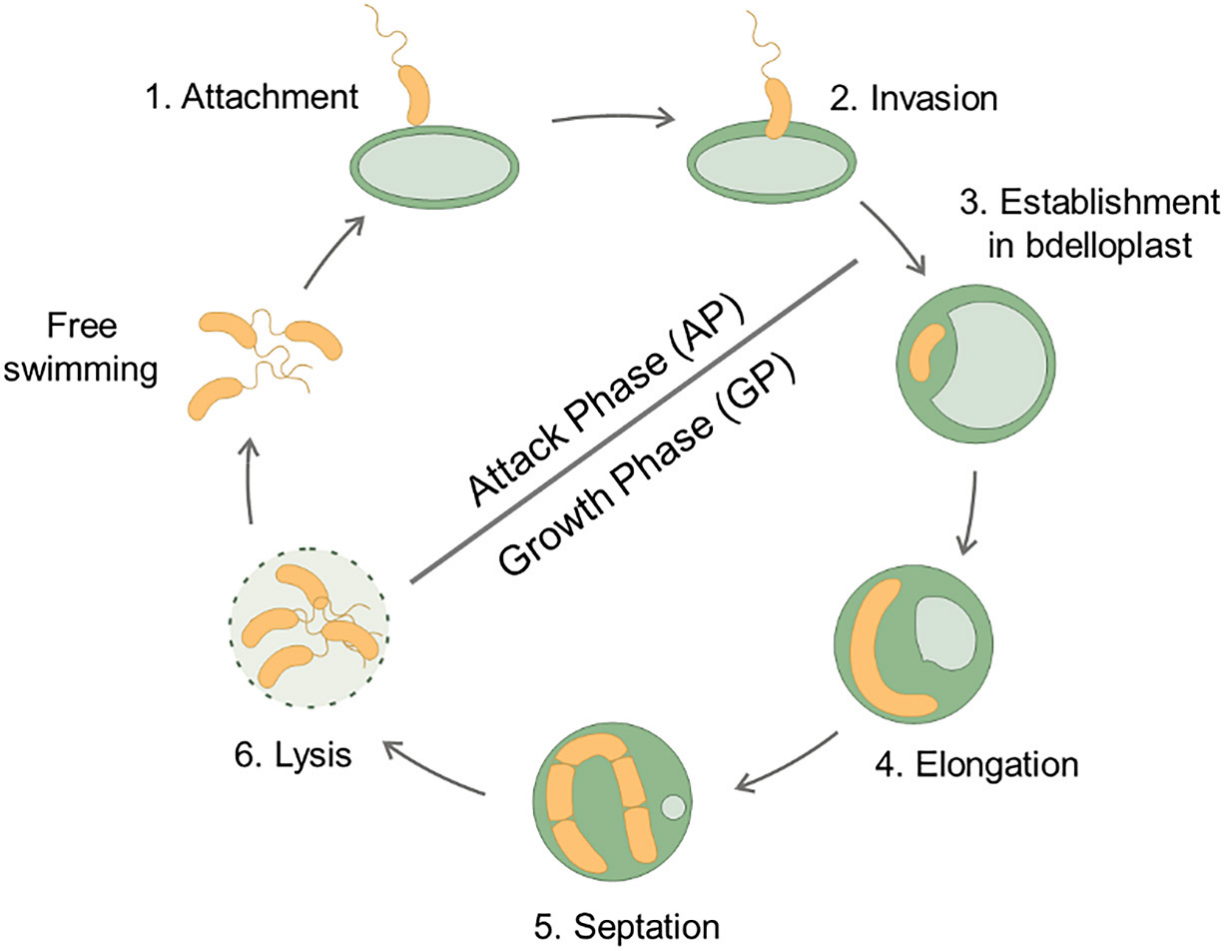
Life cycle of *B. bacteriovorus* HD100. The life cycle of *B. bacteriovorus* involves the following stages: (1) Attachment: *Bdellovibrio* attaches to the host cell, initiating infection. (2) Invasion: *Bdellovibrio* enters the prey cell's periplasm. (3) Establishment in bdelloplast: *Bdellovibrio* modifies the cell wall of the prey, leading to a rounded shape. (4) Elongation: The predator grows and replicates DNA in the bdelloplast, utilizing prey biomolecules for nutrients. (5) Septation: *Bdellovibrio* undergoes septation when resources are depleted, leading to its progeny. (6) Lysis: Progeny cells lyse the prey's cell wall, releasing into the environment to search for new prey. The complete cycle takes around 4 h (Sockett, 2009).

*Bdellovibrio bacteriovorus* is a member of the group of BALOs (*Bdellovibrio* and like organisms) (Jurkevitch, 2012). This small vibrioid‐shaped Gram‐negative bacterium is highly motile, reaching speeds up to 160 μm s^−1^ (Lambert et al., 2006). It shows a unique lifestyle comprising two phases (Figure 1): (1) a free‐living attack phase (AP), in which the predator swims to reach its prey, and (2) an intraperiplasmic growth phase (GP), that begins when the predator enters the periplasm of the prey, rounding it to generate the so‐called bdelloplast. Thanks to its vast arsenal of hydrolytic enzymes, the predator feeds, within the bdelloplast, on the products of prey degradation, elongating into a filament that eventually septates into several daughter cells to yield, on average, a progeny of 4–6 cells (Fenton, Kanna, et al., 2010). These daughter cells then lyse the prey and re‐enter the attack phase (Sockett, 2009). Although *B. bacteriovorus* was first isolated in soil (Stolp & Starr, 1963), it is ubiquitous in nature, and it can be found in terrestrial and aquatic environments, including hypersaline systems (Piñeiro et al., 2008), wastewater treatment plants (Feng et al., 2016), animal guts (Chu & Zhu, 2010; Schwudke et al., 2001) and also human microbiomes (Caballero et al., 2017; Iebba et al., 2013; Pérez‐Cobas et al., 2023). Since *B. bacteriovorus* has a wide prey range (Jurkevitch et al., 2000), it has been proposed as a “living antibiotic” to overcome the increasing multi‐drug resistant bacteria problem (Dashiff et al., 2010; Saralegui et al., 2022). Based on its predatory capacity, different applications are being developed as a method to accelerate wound healing by including the predator in hydrogels (Dwidar et al., 2012; Liu et al., 2023), as a bioagent control in agriculture (Sason et al., 2022; Saxon et al., 2014), aquaculture (Cao et al., 2018; Chen et al., 2021; Lu & Cai, 2010) and as a lytic agent for the release of valuable intracellular bioproducts, like polyhydroxyalkanoates for bioplastics production (Martínez et al., 2016). The robust hydrolytic arsenal encoded in the 3.6 Mbps genome of *B. bacteriovorus*, consisting of 150 genes encoding proteases, 10 glycanases, 15 lipases, 20 DNases and 9 RNases, positions it as a valuable enzyme reservoir with significant industrial potential (Rendulic et al., 2004). All these applications make *B. bacteriovorus* an interesting bacterium susceptible to be used as a microbial chassis (Herencias, Salgado‐Briegas, & Prieto, 2020).

A fundamental prerequisite for converting a bacterium into a microbial chassis is the availability of genetic tools for generating mutants and controlling gene expression. Synthetic biology, grounded in standardization, facilitates the consistent and predictable behaviour of biological systems, simplifying the design and assembly of genetic components to achieve specific functions. This standardization approach entails the development and application of well‐characterized biological components, including promoters, ribosomal binding sites, terminators and other regulatory elements, which can be consistently employed across various microbial chassis (Bower et al., 2010; Müller & Arndt, 2012; Shetty et al., 2008).

The first steps to convert *B. bacteriovorus* into a microbial chassis were done by Cotter and Tomashow in 1992, demonstrating that it was possible to insert a plasmid in the predator's chromosome through homologous recombination. Furthermore, they demonstrated that plasmids bearing the broad‐range origin of replication RSF1010 could replicate in *B. bacteriovorus* (Cotter & Thomashow, 1992). Homologous recombination has predominantly been employed to elucidate biological functions by creating knock‐out or knock‐in mutants. Additionally, derivative *ori* RSF1010 plasmids have been employed to complement these mutations (Capeness et al., 2013; Roschanski et al., 2011). Heterologous proteins, such as green or tomato fluorescent proteins, have been produced in *B. bacteriovorus* through knock‐in mutants and plasmid‐bearing strains (Chang et al., 2011; Fenton, Lambert, et al., 2010; Mukherjee et al., 2016; Roschanski & Strauch, 2010). Recently, the expression of heterologous genes has been carried out in *B. bacteriovorus* using pBBR1 derivative plasmids, demonstrating the replication of plasmids bearing this *ori* in the predator (Bukowska‐Faniband et al., 2020; Kaljević et al., 2021; Sathyamoorthy et al., 2021). Generally, the expression of genes in *B. bacteriovorus* has been controlled using native promoters (Capeness et al., 2013; Chang et al., 2011; Fenton, Lambert, et al., 2010; Milner et al., 2014), as well as constitutive promoters such as the kanamycin resistance gene promoter P_nptII_ (Mukherjee et al., 2016) and the synthetic constitutive promoter P_BioFab_ (Kaljević et al., 2021; Mutalik et al., 2013). The precisely controlled expression system found in *B. bacteriovorus*, which includes genes specific to the GP and AP phases (Karunker et al., 2013; Lambert et al., 2010), offers the potential to express genes during a specific growth phase. In previous work, Dwidar and Yokobayashi controlled the expression of the *mCherry* gene, encoding a red fluorescent protein, using promoters of AP‐specific genes, leading to a phase‐dependent expression. They further combined an AP‐specific promoter with the theophylline‐responsive riboswitch Theo‐F, resulting in dose‐dependent *mCherry* expression (Dwidar & Yokobayashi, 2017). However, the search for standardized constitutive and inducible promoters, which are crucial in synthetic biology, is still pending.

The hierarchical modular cloning system known as GS (Blázquez et al., 2023), based on the Golden Gate (GG) modular cloning (Weber et al., 2011), is an example of a standardized method that allows the assembly of multiple DNA fragments. Notably, the SEVA‐based GS system has been tailored for use in both model and non‐model organisms (Blázquez et al., 2023). These systems rely on the use of Type IIS restriction enzymes that cut the DNA outside the recognition site, making possible the creation of tuned sequences (fusion sites) specifically designed to allow the sequential ligation of the parts of a gene, promoter, ribosome binding site (RBS), coding sequence (CDS) and terminator, generating a complete transcription unit (TU). The high efficiency and accuracy of GS make this system suitable for characterizing genetic components.

In this work, we have developed a range of genetic tools explicitly designed for *B. bacteriovorus*. This includes a GS‐based set of destination vectors that adapt SEVA plasmids carrying the *ori* RSF1010, along with a transposon Tn7‐mediated chromosomal insertion system for achieving monocopy gene expression in the predator. Additionally, we have conducted a comprehensive characterization of synthetic constitutive and inducible promoters. These efforts expand the possibilities for tightly controlling gene expression in *B. bacteriovorus* HD100, unlocking new potential for this microbe as a versatile biotechnological platform and open the way for a multitude of opportunities, including the controlled synthesis of proteins with potential applications in co‐culture systems with two partners.

## EXPERIMENTAL PROCEDURES

### Bacterial strains, plasmids and oligonucleotides

*B. bacteriovorus*, *Escherichia coli* and *Pseudomonas putida* strains used in this work are described in Table S1. Plasmids, oligonucleotides and synthetic constitutive promoters are described in Tables S2–S4, respectively. Details regarding the molecular biology reagents can be found in the Supplementary Material.

### Media and cultivation conditions

*E. coli* and *P. putida* strains were grown in liquid or solid Lysogeny Broth LB (10 g L^−1^ NaCl, 10 g L^−1^ tryptone and 5 g L^−1^ yeast extract, pH 7.5) at 37°C and 30°C, respectively. The appropriate selection antibiotics, 50 μg mL^−1^ kanamycin (Km), 5 μg mL^−1^ or 10 μg mL^−1^ gentamicin (Gm), 75 μg mL^−1^ streptomycin (Sp), 100 μg mL^−1^ ampicillin (Ap) or 30 μg mL^−1^ chloramphenicol (Cm), were added when needed. *E. coli* BL21(DE3) Gm^R^ was grown in LB supplemented with 15 mM glucose, to increase biomass production, and 10 μg mL^−1^ Gm when used as prey.

*B. bacteriovorus* HD100 was co‐cultured with *P. putida* KT2440 or *E. coli* BL21(DE3) at OD_600_ 1 as preys as previously described in Herencias et al. (2017). *B. bacteriovorus* cell viability was determined in co‐cultures following the double‐layer method (Herencias et al., 2017).

For *B. bacteriovorus* HD100 conjugation experiments, PYE plates (10 g L^−1^ peptone, 3 g L^−1^ yeast extract and 1.5% agar) were used. The selection of *B. bacteriovorus* transconjugants was performed on DNB^+^ plates following the double‐layer method. *E. coli* BL21(DE3) transconjugants were selected in M9 minimal medium plates (2 mM MgSO_4_, 0.1 mM CaCl_2_, 12.8 g L^−1^ Na_2_HPO_4_ · 7H_2_O, 3 g L^−1^ KH_2_PO_4_, 0.5 g L^−1^ NaCl, 1 g L^−1^ NH_4_Cl, 2% w/v glucose and 1.5% w/v agar). *P. putida* KT2440 transconjugants were selected on cetrimide plates (Sigma‐Aldrich, USA) prepared according to the manufacturer's indications and supplemented with 10 μg mL^−1^ Gm. *E. coli* DH5α, *E. coli* DH5α λ*pir* ^+^, *E. coli* DH10B and *E. coli* CC118 λ*pir* ^+^ were used as hosts for gene cloning. *E. coli* HB101, bearing the pRK600 plasmid, was used as a helper strain for conjugation experiments. *E. coli* BL21(DE3) and *P. putida* KT2440 wild‐type and derivative strains were used as prey for *B. bacteriovorus* strains cultivation.

### Construction of GS compatible plasmids

To develop a modular and extensible system specially designed for *B. bacteriovorus*, we adapted the hierarchical assembly cloning technique GS described for both model and non‐model Gram‐negative bacteria (Blázquez et al., 2023). GS is based on the combination of the Level 0 parts essential for gene expression, that is, promoter, RBS, CDS and terminator, to form a complete Level 1 TU. Different TUs can be combined in a Level 2 multigene construct. Levels 1 and 2 assemblies can be applied to heterologously express genes in the receptor chassis. The selection of the alternative levels during the assembly process is based on the antibiotic markers included in the plasmids of each level. In this sense, it is worth noting that *B. bacteriovorus* is sensitive to Km and Gm, applied for Levels 1 and 2 in this work, with MICs of 0.7 μg mL^−1^ and 0.06 μg mL^−1^, respectively (Marine et al., 2020).

In our approach, we have chosen two SEVA plasmids containing the RSF1010 *ori* (pSEVAX5X) as backbones, named pSEVA251 and pSEVA651, that contain Km and Gm resistance genes, respectively (Martínez‐García et al., 2023). Since these plasmids have two recognition sites of the enzyme BpiI that would decrease the efficiency of GS reactions, they were first domesticated to eliminate these sites (Marillonnet & Werner, 2015), using the FastCloning protocol (Li et al., 2011). The pSEVA251 plasmid was amplified with the oligonucleotide pairs SS61–SS64 and SS62–SS63 (Table S3), generating two overlapping DNA fragments. Both fragments were mixed with a 1:2 vector–insert ratio, where insert refers to the shortest DNA fragment. This mix was then digested with DpnI in 1X CutSmart buffer for 1 h at 37°C to eliminate the template plasmid. Subsequently, 5 μL of the digestion was used to transform 100 μL of chemically competent *E. coli* DH10B cells. Transformed cells were selected on LB plates supplemented with Km. Plasmids were purified from five different colonies and checked by BpiI digestion. A similar protocol was followed to domesticate the pSEVA651 plasmid, using LB plates supplemented with Gm to select transformant cells. The domestication resulted in the plasmids pSEVA251‐dom and pSEVA651‐dom (Figure S1A).

The cargoes of the GS plasmids pSEVA2319[g2] and pSEVA6319[gE], encoding the *lacZα* fragment for α‐complementation of the β‐galactosidase, were digested with PacI/SpeI and ligated into the domesticated plasmids, yielding the plasmids pSEVA2519[g2] and pSEVA6519[g2] for Level 1 reactions and pSEVA2519[gE] and pSEVA6519[gE] for Level 2 reactions (Figure S1A). For transposon Tn7‐mediated chromosomal integration, the cargoes of pSEVA2319[g2] and pSEVA6319[gE] were digested with PacI/SpeI restriction enzymes and ligated into the pTn7‐M plasmid, resulting in the pTn7‐19[g2] and pTn7‐19[gE] plasmids, for Level 1 and Level 2 reactions, respectively (Figure S1B).

### GS reactions

GS reactions were conducted according to the protocol published for GG reactions (Manoli et al., 2023). After the GS reactions, 100 μL aliquots of chemically competent *E. coli* DH5α λ*pir* ^+^ were heat shock transformed with 5 μL of the reaction products. Transformed cells were plated on LB‐agar supplemented with Sp (for Level 0), Km (for Level 1), Gm (for Level 2), or Km and Gm (for Tn7 constructions), 0.5 mM IPTG and 40 μL mL^−1^ X‐gal and grown overnight at 37°C for selection of the disruption of α‐complementation β‐galactosidase activity. Several white colonies per transformation were transferred to LB medium for plasmid purification. The extracted plasmids were confirmed by sequencing with primers RK81 and RK82 (for Level 0), PS1 and PS2 (for Level 1 and Level 2), or PS1, PS2 and pBG‐Fwd (for Tn7 constructions). See Table S3 in the Supplementary Material for details.

### Conjugation procedures

Recombinant *B. bacteriovorus* HD100 strains were obtained by tri‐ or tetraparental matings. The recipient strain (*B. bacteriovorus* HD100) was co‐cultured in Hepes^+^ for 24 h, using *E. coli* BL21(DE3) as prey, and filtered twice with a 0.45 μm pore filter (Sarstedt, Germany). Each donor strain (*E. coli* DH5α λ*pir* ^+^ bearing the Level 1 or Level 2 plasmid), and the helper strain (*E. coli* HB101 [pRK600]), was cultured overnight in LB supplemented with the corresponding antibiotic. For tetraparental matings, the transposase‐leading strain (*E. coli* DH5α [pTnS‐1]) was included. From each culture, 1 mL was pelleted, washed with 0.85% (w/v) NaCl solution and mixed in a final volume of 200 μL of PYE medium. This mix was placed on a 0.22 μm nitrocellulose filter over a PYE plate and incubated overnight at 30°C. Each conjugation product was resuspended in 1 mL of Hepes^+^, and serial dilutions were plated following the double‐layer method in DNB^+^ plates supplemented with Gm (10 μg mL^−1^ for replicative plasmids and 5 μg mL^−1^ for Tn7 chromosomal integrations) in both layers using *P. putida* KT2440 Gm^R^ as prey (see below). The plates were incubated at 30°C for 3–4 days until plaques were visible. Ten plaques of each construction were transferred to liquid DNB^+^ supplemented with the same antibiotic and prey. The predated co‐cultures were filtered with 0.45 μm filters to eliminate remaining donor cells and propagated again in fresh DNB^+^ with the prey. To maintain the plasmids, the antibiotic was always added to co‐cultures. The plasmid‐bearing strains were checked by plasmid isolation of a 16 mL co‐culture (filtered twice with 0.45 μm filters), and they were subsequently sequenced with the primers PS1 and PS2 (Table S3), which anneals downstream and upstream of the GS core, respectively. The chromosomal insertion recombinants were checked by PCR amplification using 1 μL of the co‐cultures in a final volume of 25 μL, with the primers SS147 and SS126 (Table S3), which anneals in the genome of *B. bacteriovorus* HD100 (Figure S2). The amplicons were further sequenced with the primers SS204 and SS205 (Table S3), which anneals within the amplicon region (Figure S2).

To calculate the efficiency of conjugations, the mating results were also plated following the double‐layer method in DNB^+^, without Gm, using *P. putida* Gm^R^ as prey and incubated until lysis plaques were visible. The efficiencies of conjugations were calculated with the ratio of Gm^R^ *B. bacteriovorus* HD100 cells and the total *B. bacteriovorus* HD100 cells.

Tetraparental matings were conducted with *E. coli* BL21(DE3) and *P. putida* KT2440 to obtain stable antibiotic‐resistant strains. The Level 1 construction pTn7‐19[g2]‐ncTU (Table S7) was chromosomally integrated into *E. coli* BL21(DE3) and *P. putida* KT2440 following the protocol described above. *E. coli* BL21(DE3) transconjugants were selected in plates of M9 medium supplemented with Gm, 0.5 mM IPTG and 40 μL mL^−1^ X‐gal. *P. putida* KT2440 transconjugants were selected in cetrimide plates supplemented with Gm.

### Construction of a library of strains expressing different levels of a reporter gene

**FIGURE 2:**
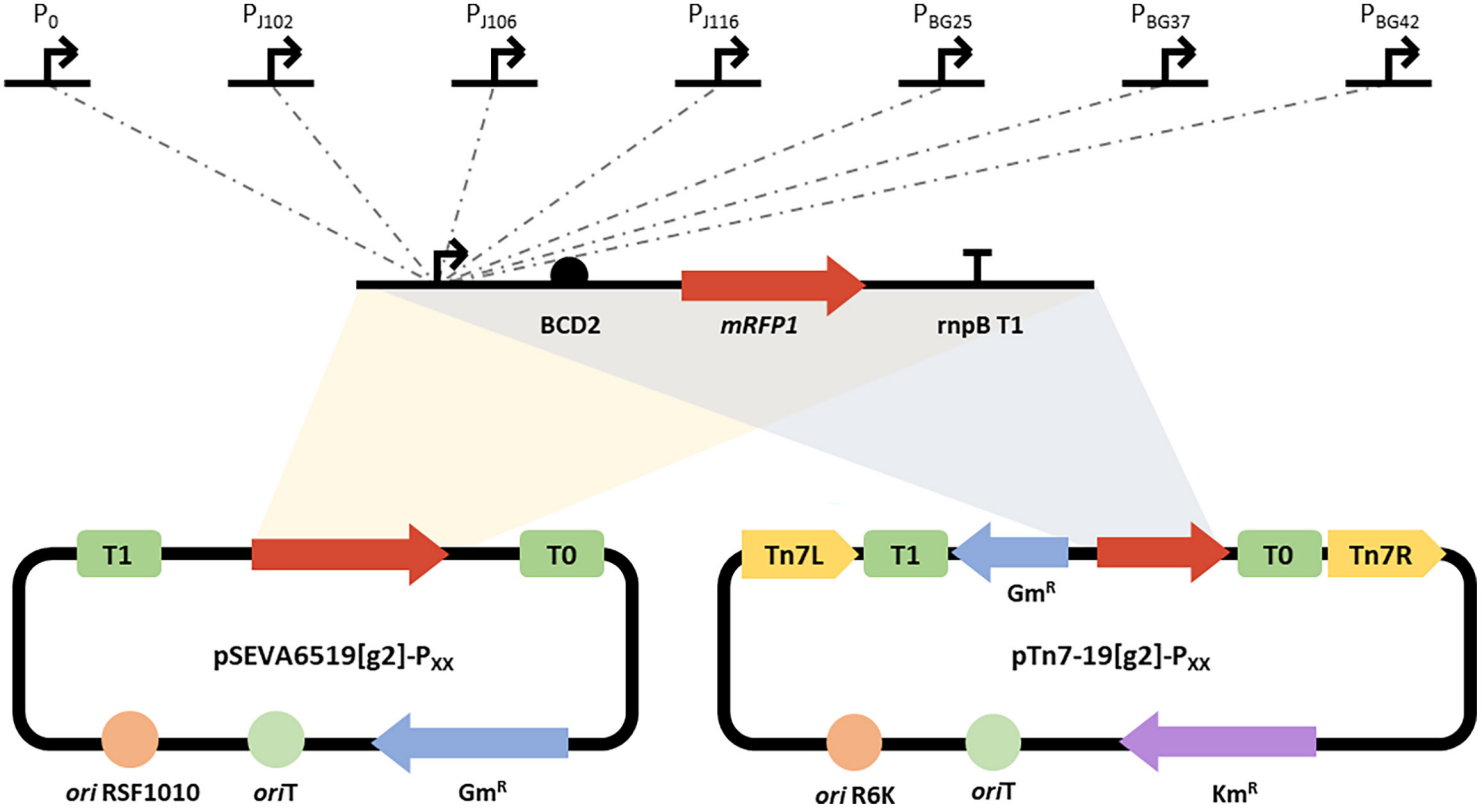
mRFP1 expressing TUs combining the selected set of promoters. The selected promoters were ligated to the synthetic translational coupler BCD2, the mRFP1 and the rpnB_T1 terminator through a GS Level 1 reaction, using pSEVA6519[g2] and pTn7‐19[g2] as destination vectors. GS assemblies are detailed in Table S4.

Six Level 1 constructions were made with the adapted vectors pSEVA6519[g2] and pTn7‐19[g2] as backbones for the non‐chromosomal and Tn7‐mediated chromosomal expression, respectively, through GS reactions see above. The gene of the monomeric Red Fluorescent Protein *mRFP1* (Campbell et al., 2002) was cloned under the control of six synthetic constitutive promoters of different strengths: three were selected from the “Anderson collection” (https://parts.igem.org/Promoters/Catalog/Anderson) and three from the “Zobel collection” (Zobel et al., 2015). These TUs were completed with the synthetic bicistronic RBS BCD2 (Mutalik et al., 2013), and the rnpB_T1 terminator (Cambray et al., 2013), to evaluate the different levels of mRFP1. Two additional Level 1 reaction was done with the pL0‐ncTU‐AI and the pL0‐P_0_‐AB plasmids to obtain negative controls (Figure 2).

Additionally, five Level 1 TUs were generated cloning the gene of the monomeric and superfolder Green Fluorescent Protein *msfGFP* (Zobel et al., 2015) under the control of five inducible promoters (Figure S3). These TUs were completed with the BCD2 translational coupler and the rnpB_T1 terminator. Thes Level 1 TUs were combined with their respective Level 1 TU containing the regulator for each inducible promoter to form a Level 2 construction, using the plasmid pTn7‐19[gE] as backbone.

The GS constructions were confirmed by sequencing with primers RK81 and RK82 for Level 0 (Tables S3 and S6), PS1 and PS2 for Level 1 and Level 2 (Table S3), or PS1, PS2 and pBG‐Fwd for Tn7 constructions (Table S3).

The plasmids with these Level 1 and Level 2 constructions (see Table S7, Supplementary Material for details) were transferred to *B. bacteriovorus* HD100 through tri‐ or tetraparental mating. The recombinant strains were verified by plasmid isolation or, in the case of the chromosomal Tn7‐mediated integration, by PCR amplification and subsequent sequencing.

### Stability of recombinant strains

To check the stability of the recombinant strains *B. bacteriovorus* HD100 [pSEVA6519[g2]‐ncTU] and Bd08‐ncTU, co‐cultures of *E. coli* BL21(DE3) Gm^R^ and each strain were incubated at 200 rpm and 30°C, propagating 100 μL every 24 h in fresh *E. coli* BL21(DE3) Gm^R^ suspension for 10 times without antibiotic pressure. Co‐cultures were plated following the double‐layer method, both with and without Gm supplementation, to measure Gm^R^ cells and total viable cells of *B. bacteriovorus* recombinant strains.

### Promoters' characterization

To ensure homogeneous populations, 3 rounds of co‐cultivation with *E. coli* BL21(DE3) Gm^R^ suspensions were performed in shake flasks. Then, the promoters' strengths from the different *B. bacteriovorus* recombinant strains were analysed in fresh co‐cultures. When inducible promoters were evaluated, the respective inducer (Table S5) was added at the onset of these co‐cultures.

Predator cell viability was quantified at time 0 following the double‐layer method. Subsequently, 200 μL of each co‐culture was loaded into a well in 96‐well plates (Nunc™ Nunclon Multi‐Dishes, Thermo Fisher Scientific, USA). OD_600_ and fluorescence intensity of the co‐cultures were measured every 20 min for 24 h with a VICTOR Nivo (PerkinElmer, USA) microplate reader with a double orbital motion at 600 rpm. Red fluorescence was measured using an excitation filter of 580/20 nm and an emission filter of 625/30 nm. Green fluorescence was measured using an excitation filter of 480/30 nm and an emission filter of 530/30 nm. Since the growth of *B. bacteriovorus* cannot be followed by OD_600_, the predation efficiency of each strain was inferred by the decrease in OD_600_ caused by prey predation and absolute fluorescence data were used for the characterization of the promoters. The features of the curves were extracted using the R package “CuRveR” by fitting the data to an adapted generalized sigmoid curve based on the Richard equation (Kaljević et al., 2021; Remy et al., 2022):

(1) $$P\left(t\right)=p_{\min}+\frac{p_{\max}-p_{\min}}{1+e^{\frac{4r_{\max}\left(t-s\right)}{p_{\min}-p_{\max}}}}$$

In this formula, $P\left(t\right)$ represents the population at time t measured in terms of OD_600_ or fluorescence units. $p_{\min}$ indicates the minimum population, serving as the lower asymptote and measured in OD_600_ or fluorescence units. $p_{\max}$ corresponds to the maximum population, acting as the upper asymptote and measured similarly. The parameter $r_{\max}$ signifies the maximum rate, and it is positive if the population is growing (indicating the growth rate) and negative if the population is declining (indicating the killing rate). These rates are measured in h^−1^. The variable s represents the inflection point, denoting the time when the rate reaches an extremum, measured in h.

Single‐cell fluorescence was analysed with Cytek Aurora flow cytometer (5‐laser; 355 nm, 405 nm, 488 nm, 561 nm and 640 nm, Cytek Biosciences, USA), using the SpectroFlo Software v2.2.0.2. For each sample, 50,000 total events were recorded. This technique was used to evaluate the homogeneity of the mutant populations, intensity of the fluorescence, and to quantify the cell number of predated co‐cultures. Data processing was performed using FlowJo v10.9 (BD, USA).

The inducible promoters were compared using the Hill function (Ang et al., 2013). The median fluorescence intensities at each concentration (obtained through flow cytometry) were fitted using OriginPro 2022b (OriginLab, USA) to the Hill equation (Equation 2):

(2) $$y=k^{\prime }+k\left(\frac{x^{n}}{K^{n}+x^{n}}\right)$$

where y represents the concentration of the expressed protein (measured as fluorescence intensity), $k^{\prime }$ is the basal production rate, k is the maximum additional production rate resulting from upregulation and x is the concentration of the inducer.

### Fluorescence microscopy

Cell cultures were observed using a Leica DM4B epifluorescence microscope (Leica, Germany) with a 100X phase‐contrast objective. Images were captured using a Leica DFC345 FX camera. For mRFP1 and MsfGFP visualization, Y3 and L5 filter systems were employed, respectively. Before adding each sample, microscope slides were coated with a thin layer of 0.1% poly‐L‐lysine to immobilize cells.

### Statistical analysis

Experiments were conducted in duplicate or triplicate. The error bars or the filled areas on the graphs indicate the standard deviation among the replicates. Normality and homoscedasticity of the samples were tested using the Shapiro–Wilk normality test and the Bartlett's test, respectively. For normal and homoscedastic samples, an analysis of variance (ANOVA, *α* = 0.05) test was conducted to compare three or more data sets, followed by the Tukey post‐hoc test or the Bonferroni test when significant differences were detected. For normal and heteroskedastic sample comparison, the Brown–Forsythe ANOVA (*α* = 0.05) test was performed. Paired Student's *t*‐tests (*α* = 0.05) were used for the comparison of two populations. Statistical analyses were done with R and GraphPad Prism 9 (GraphPad Software, Inc., USA).

## RESULTS

### Adapting the GS system to *B. bacteriovorus* HD100

Extra‐chromosomal DNA bearing the broad‐range RSF1010 and pBBR1 *oris* can replicate in *B. bacteriovorus* (Bukowska‐Faniband et al., 2020; Cotter & Thomashow, 1992; Roschanski & Strauch, 2010). To expand the genetic tools for *B. bacteriovorus*, the Level 1 and 2 from the GS system were adapted to allow heterologous gene expression in *B. bacteriovorus* HD100 using replicative plasmids.

Two SEVA plasmids with the RSF1010 *ori* (pSEVAX5X), pSEVA251 and pSEVA651, containing resistance genes for Km and Gm, respectively (Martínez‐García et al., 2023), were chosen as backbones. These plasmids were domesticated to eliminate the BpiI recognition sites, resulting in the plasmids pSEVAX51‐dom (Figure S1A). Afterwards, the GS Level 1 position 2 and Level 2 position E cores were subcloned into the PacI/SpeI site of these two domesticated plasmids, resulting in four plasmids (Figure S1A). The adapted plasmids pSEVA2519[g2] and pSEVA6519[g2] allow the combination of GS Level 0 parts to generate complete Level 1 TUs able to be expressed in *B. bacteriovorus* HD100. The Level 2 plasmids pSEVA2519[gE] and pSEVA6519[gE] allow the expression of multigene constructs in this host. Since the adapted plasmids are essentially GS plasmids, the same nomenclature has been followed: pSEVAXYZ, where X is the antibiotic, Y is the origin of replication (RSF1010), and Z is the cargo (Martínez‐García et al., 2023). In this case, the cargo has been numbered as 19[gP], where P is the GS position (2 or E). The inclusion of the *lacZα* gene between the two Type IIS enzyme's recognition sites allows the blue/white screening through the β‐galactosidase α‐complementation since the *lacZα* gene is lost when a correct assembly has been performed (Blázquez et al., 2023). These GS‐adapted plasmids increase the possibilities to modify *B. bacteriovorus* HD100 in a transient manner using replicative plasmids.

### *att*Tn7 site in the genome of *B. bacteriovorus* HD100

Replicative plasmids are valuable tools for heterologous gene expression when increased gene copies are required for higher expression levels. Nevertheless, a stable chromosomal integration eliminates the requirement of maintaining a selective pressure such as antibiotics addition. In this sense, the Tn7 transposon system has been demonstrated to be a powerful tool to integrate a single copy in the chromosome of many bacteria (Bordi et al., 2008; Choi & Schweizer, 2006; Lambertsen et al., 2004). The Tn7 transposon integrates a single copy of the mobile element in a specific chromosomal region called *att*Tn7, located downstream of the glutamine‐fructose‐6‐phosphate aminotransferase (*glmS*), a gene related to the cell wall synthesis and widespread among bacteria kingdom (Parks & Peters, 2007). The transposition of the mobile element is triggered by the protein TnsD, which binds to a region located at 3′ of the *glmS* gene (Figure S2A). The critical nucleotides conserved in bacteria where the Tn*7* transposon's mobile element can integrate (Mitra et al., 2010) are also conserved in the *glmS* gene of *B. bacteriovorus* HD100, located between positions 3,335,698 and 3,337,584 on the reverse strand (Figure S2B) (Rendulic et al., 2004). Therefore, we hypothesize that the Tn7‐mediated chromosomal integration will be feasible in this bacterium, enabling its stable genetic modification. As a proof of concept, the mobilizable element of the pTn7‐M (Zobel et al., 2015), containing the Gm‐resistance gene, was integrated into *B. bacteriovorus* HD100 through a tetraparental mating. Four *B. bacteriovorus* Gm^R^ colonies were checked to verify the genomic insertion: the *glmS* surrounding region was amplified and sequenced, confirming the insertion point *att*Tn7+0 at the guanine 3,335,765 on the forward strand in the genome of *B. bacteriovorus* HD100.

### Construction of mRFP1‐producing strains

Several experiments have been carried out using native promoters of *B. bacteriovorus*, mainly to complement mutated genes (Avidan et al., 2017; Roschanski et al., 2011), but also to synthesize heterologous proteins such as the red fluorescent protein mCherry using AP‐specific promoters (Dwidar & Yokobayashi, 2017). However, only some commonly used promoters, such as the Km resistance gene promoter *P*_nptII_, have been tested in *B. bacteriovorus* to express heterologous genes (Mukherjee et al., 2016). The use of heterologous expression systems offers the possibility of decoupling the expression to native regulation that remains unknown in most non‐model organisms. The feasibility of applying heterologous promoters strongly depends on the transcription machinery's compatibility, which is mainly orchestrated by σ factors that bind to the DNA and the RNA polymerase (Helmann, 2019). In this sense, *B. bacteriovorus* possesses an RpoF/FliA (gene *bd3318*) σ factor that controls the transcription of about two‐thirds of the AP genes (Karunker et al., 2013). According to Lambert et al. (2012), aside from the RpoF/FliA σ factor, the genome of *B. bacteriovorus* contains other predicted σ factors: an RpoD σ^70^ encoded by the gene *bd0242*, an RpoD homologue encoded by the gene *bd3314*, an RpoN‐like σ factor encoded by the gene *bd0843* and two RpoE‐like σ factors encoded by the genes *bd0743* and *bd0881* (Lambert et al., 2012). An in silico analysis revealed that RpoD (Bd0242) is composed by four domains (Figure S4), including the domain 1.1, which is essential for inhibiting the DNA binding of free σ^70^ factors and, therefore, can be classified as housekeeping group I σ factor (Gruber & Gross, 2003). Despite sharing only 55.7% similarity with *E. coli* K‐12's RpoD, the subdomains responsible for DNA binding exhibit higher similarities. Specifically, subdomains 2.4 and 4.2, which recognize −10 and −35 boxes, share 100% and 96.6% similarity, respectively (see Figure S4). Therefore, we could hypothesize that the σ^70^‐dependent promoters for *E. coli* should also be recognized for the RpoD of *B. bacteriovorus* HD100.

To establish a panel of constitutive promoters for tightly controlling the synthesis of heterologous gene, we tested the expression of the gene *mRFP1* under the regulation of six σ^70^ synthetic promoters that show different strengths in model organisms such as *E. coli* or *P. putida* (Table S4). Since this work aimed to characterize these promoters in *B. bacteriovorus* HD100, we kept constant the other parts that compose each TU, that is, the RBS and the terminator. We combined the Level 0 plasmids containing the different parts with the adapted destination vectors pSEVA6519[g2] and pTn7‐[g2] (Figure S1), obtaining six TUs for each destination vector, for plasmid expression and for Tn7‐mediated chromosomal integration (Figure 2). Along with the synthetic promoters, we engineered promoter‐less constructs by including the non‐functional promoter sequence (pL0‐P_0_‐AB) at the promoter position and empty vectors fusing a non‐coding TU sequence (pL0‐ncTU‐AI) to the destination vectors. The plasmids with these constructions were transferred to *B. bacteriovorus* HD100 by tri‐ or tetraparental matings depending on whether they are replicative or integrative plasmids, respectively. Since none of these conjugation procedures had been previously tested in *B. bacteriovorus*, we measured the transfer efficiency for each construction. As can be seen in Table 1, the transfer frequency of the replicative plasmid expressed as plaque‐forming units (PFUs) of Gm^R^ *B. bacteriovorus* HD100 per total PFUs of *B. bacteriovorus* HD100 ranged from 10^−7^ to 10^−3^, whereas the Tn7 chromosomal insertion frequency was 10^−6^ in every construction.

**TABLE 1:** Transfer frequency of constructed plasmids.

Plasmid	Transfer frequency^a^	Plasmid	Insertion frequency^a^
pSEVA6519[g2]‐ncTU	1.38 × 10^−3^	pTn7‐19[g2]‐ ncTU	2.99 × 10^−6^
pSEVA6519[g2]‐P_J116_‐R	9.84 × 10^−4^	pTn7‐19[g2]‐P_J116_‐R	1.05 × 10^−6^
pSEVA6519[g2]‐P_J102_‐R	2.78 × 10^−4^	pTn7‐19[g2]‐P_J102_‐R	1.75 × 10^−6^
pSEVA6519[g2]‐P_J106_‐R	1.53 × 10^−3^	pTn7‐19[g2]‐P_J106_‐R	1.20 × 10^−6^
pSEVA6519[g2]‐P_BG37_‐R	1.94 × 10^−3^	pTn7‐19[g2]‐P_BG37_‐R	1.15 × 10^−6^
pSEVA6519[g2]‐P_BG42_‐R	3.63 × 10^−7^	pTn7‐19[g2]‐P_BG42_‐R	1.60 × 10^−6^
pSEVA6519[g2]‐P_BG25_‐R	2.47 × 10^−4^	pTn7‐19[g2]‐P_BG25_‐R	1.78 × 10^−6^

^a^ Transfer/insertion frequency: PFUs of Gm^R^ *B. bacteriovorus* HD100 per total PFUs of *B. bacteriovorus* HD100. The number of recombinants was determined in double‐layer DNB^+^ plates without supplementation and supplemented with 5 μg mL^−1^ Gm, using *P. putida* Gm^R^ as prey in both plates.

**FIGURE 3:**
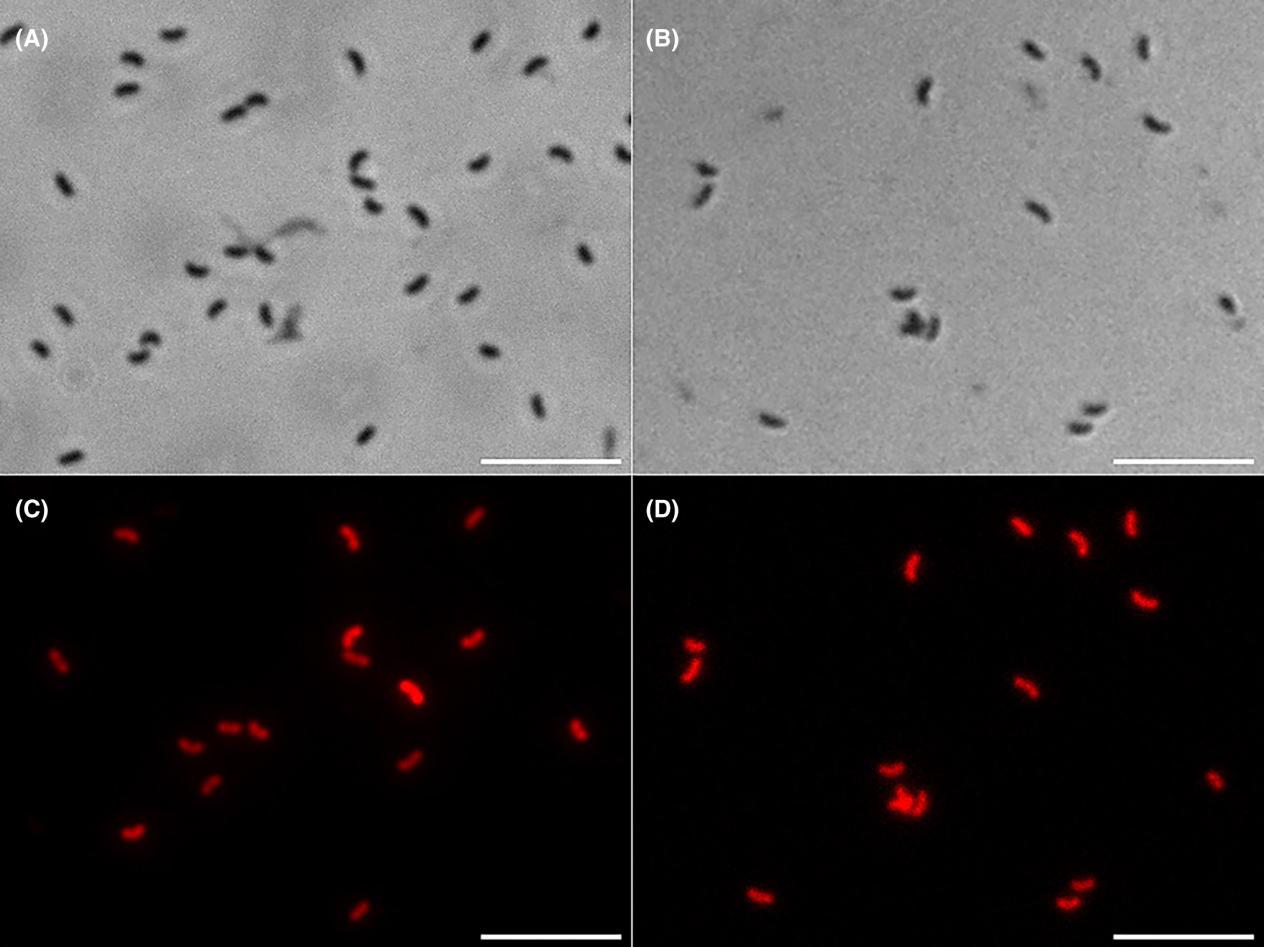
Phase‐contrast and fluorescence microscopy images of mRFP1‐producing *B. bacteriovorus* recombinant strains. Pictures of 24‐h co‐cultures in Hepes^+^ supplemented with 5 μg mL^−1^ Gm and *E. coli* BL21(DE3) Gm^R^ as prey. (A) and (C) Co‐culture of a plasmid‐bearing strain. (B) and (D) Co‐cultures of a Tn7‐integrated strain. Scale bars correspond to 5 μm.

When the harbouring plasmid strains were grown with Gm pressure (5 μg mL^−1^), the populations showed heterogeneity in terms of fluorescent phenotype: some cells accumulated very high levels of mRFP1, whereas others did not (Figures 3A,C and S5). In contrast, the strains that synthesized the mRFP1 from the chromosomal insertion showed homogeneous populations (Figure 3B,D).

To confirm the stability of both strategies, we performed two serial co‐cultures without selective pressure. The control strains *B. bacteriovorus* HD100 [pSEVA6519[g2]‐ncTU] and Bd08‐ncTU were grown in a suspension of *E. coli* BL21(DE3) Gm^R^ at OD_600_ 1 in Hepes^+^ without Gm. The 24‐h lysed co‐cultures served as inoculum for fresh prey suspensions without Gm. This successive co‐culture was repeated for 10 days. Gm^R^‐resistant and total viable cells of the recombinant predators were measured daily (Figure S6). After nine propagations, Gm^R^‐resistant cells were significantly lower in the HD100 [pSEVA6519[g2]‐ncTU] strain. In contrast, no differences were observed in the chromosomal recombinant.

The plasmid instability and the *mRFP1* heterogeneity expression were also confirmed by flow cytometry (Figure S7). When the recombinant strains were grown without antibiotic pressure, the non‐fluorescent events increased after 24 h because of plasmids lost. In contrast, in the presence of 5 μg mL^−1^ Gm, we observed that the non‐fluorescent population remained constant, but two modal peaks could be observed, meaning that there are two populations expressing different levels of *mRFP1*. When the concentration of Gm was increased to 10 μg mL^−1^, the smaller peak shifted to the right, appearing two peaks only in the strain bearing the construction with the promoter P_J116_. However, the baseline of the positive peaks expanded throughout the edge, suggesting the presence of a heterogeneous population.

### Characterization of synthetic constitutive promoters

**FIGURE 4:**
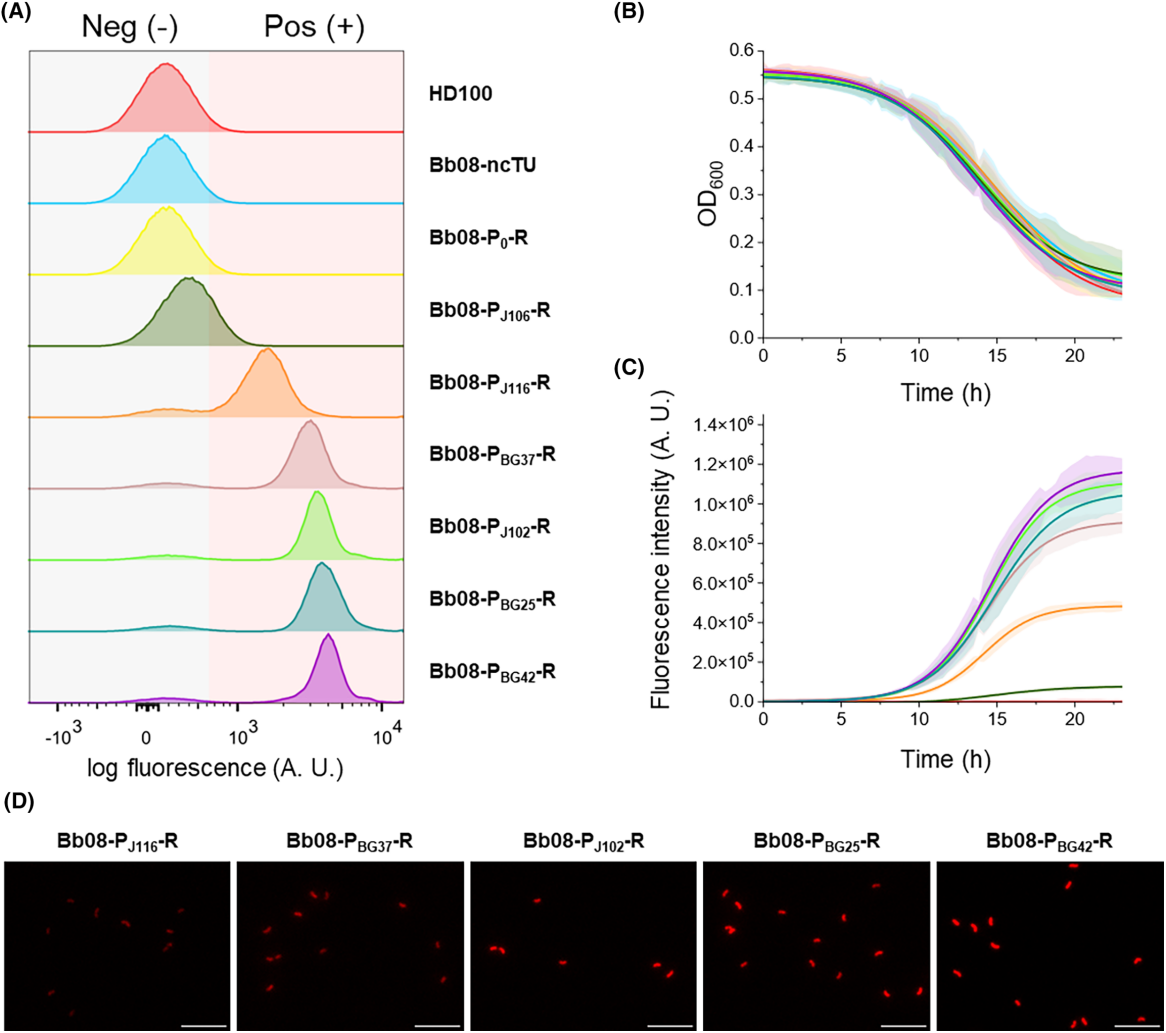
Characterization of synthetic constitutive promoters. (A) Single‐cell fluorescence analysis of red fluorescent *B. bacteriovorus* strains. The strains were grown in E. coli BL21(DE3) Gm^R^ suspensions at OD_600_ 1 in Hepes^+^. At 24 h, the predated co‐cultures were analysed by flow cytometry, using *B. bacteriovorus* HD100 as negative control. Three biological replicates were concatenated in each sample to make the stacked histogram. (B) Absorbance curves of the prey, after blank subtraction (Hepes^+^). Points were fitted to a Richard curve using CuRveR. (C) Fluorescence intensity curves of each strain, after blank subtraction (HD100 strain). Points were fitted to a Richard curve using CuRveR. (D) Fluorescence microscopy images. Scale bars represent 5 μm.

To fine‐tune gene expression in *B. bacteriovorus* HD100, the strength of the promoters needs to be characterized since these orthogonal tools do not always behave similarly in non‐related species. Considering the results presented above in terms of population homogeneity, strains with constructions inserted at the *att*Tn7 site were chosen for these assays. Recombinant predators were co‐cultured with *E. coli* BL21(DE3) Gm^R^ suspension in Hepes^+^ for 24 h, and they were single‐cell analysed by flow cytometry (Figure 4A). Every co‐culture showed a typical unimodal fluorescence curve, indicating homogeneity in the fluorescent population. To compare the expression of the different promoters in *B. bacteriovorus* HD100, the median of the expression of each promoter was normalized by the highest expression promoter P_BG42_ (Table S8). The promoters were normalized to the lowest expression promoter, P_J106_, to determine the expression range, resulting in a relative range of 9.01 ± 0.43‐fold.

Growth phase‐dependent nature of the promoters was confirmed following the strain's growth in a 96‐well microplate reading, registering both OD_600_ and fluorescence intensity for 24 h. To standardize inoculation with a consistent range of *Bdellovibrio* cells, the total 24‐h co‐culture events were assessed using flow cytometry, confirming that all cultures had statistically the same range of total events (from 9.27 ± 0.08 to 9.33 ± 0.1 Log events mL^−1^, Table S8). The initial viable cells of each strain were also calculated following the double‐layer method, resulting in statistically the same initial concentration (from 7.26 ± 0.02 to 7.35 ± 0.06 Log PFUs mL^−1^, Table S8).

The absorbance curves of the co‐cultures (Figure 4B) show a decrease in OD_600_, which corresponds with the prey's death. This decline facilitates the comparison of predation effectiveness among the different co‐cultures. In all the co‐cultures, the predation took place between 5 and 20 h, mainly being maximal at around 14 h for all the strains. Although there were slight variations in the maximal prey death rate (*r*_max_) and the times when the predation rate is maximal (s) (see Table S8), these differences did not reach statistical significance. This implies that predation was not affected by the expression of *mRFP1* and that the *s* value of the fluorescence curves is an optimal measure for controlling *Bdellovibrio*'s growth.

As shown in Figure 4C, the fluorescence intensities of each strain showed a consistent trend. Over time, the fluorescence intensity increases following a typical microbial growth curve, suggesting a positive correlation with the number of *Bdellovibrio*'s cells. Moreover, the fluorescence intensity reached a plateau when the co‐cultures entered the attack phase. Hence, the set of promoters showed a constitutive behaviour that was further confirmed by following the fluorescence intensities of the co‐cultures, which exhibited a slow steady decrease (data not shown).

In this sense, flow cytometry findings were validated by fluorescence microscopy, observing different red fluorescence intensities in the recombinant strains (Figure 4D). As it is shown in Figure 4A, Bb08‐P_J106_‐R displayed a very low fluorescence intensity. Consequently, it was omitted in Figure 4D since it could be hardly differentiated from the negative control.

### Controlling gene expression through inducible promoters

The use of inducible promoters is an established method to elucidate biological functions of proteins, and they are also essential to tightly control the expression of genes, both native and heterologous, that can be toxic for the host (Goldstein & Doi, 1995). Despite the possibilities that *B. bacteriovorus* offers as a microbial chassis, there is a lack of inducible promoters to control gene expression. Inducible promoters such as P_lac_ and P_taclac_ have been tested in *Bdellovibrio*, but they did not show an increased response when their respective inducers were applied (Flannagan et al., 2004; Roschanski & Strauch, 2010). A pioneer work by Dwidar and Yokobayashi opened the possibility of controlling gene expression in *B. bacteriovorus* HD100 through synthetic riboswitches (Dwidar & Yokobayashi, 2017).

In this work, we have tested a battery of inducible promoters of diverse natures whose activity has been demonstrated in model organisms. On the one hand, we chose carbohydrate inducible regulator/promoter systems such as P_BAD_/AraC (Guzman et al., 1995) and P_rhaBAD_/RhaSR (Haldimann et al., 1998). On the other hand, the regulator/promoter systems induced by small diffusible molecules such as P_JExD_/EliR (Ruegg et al., 2018), P_m_/XylS (Gawin et al., 2017) and P_b_/ChnR (Steigedal & Valla, 2008).

Taking advantage of GS, we generated Level 2 assemblies combining each Level 1 construct, including the promoter tested (i.e., the promoter of interest fused to the synthetic bicistronic RBS BCD2, the *msfGFP* CDS and the rnpB_T1 terminator), with its respective Level 1 regulator construct (Table S7 and Figure S3). The Level 1 regulator assemblies for each promoter/regulator system were obtained from the GS Collection when available and otherwise constructed. Considering the results presented above, we chose the pTn7‐[gE] as the destination vector to allow the integration into the chromosome of *B. bacteriovorus* HD100 at the *att*Tn7 site. Five Level 2 constructions were obtained, one for each pair regulator/promoter (Figure S3). These constructions were conjugated to *B. bacteriovorus* HD100 following the tetraparental procedure.

*B. bacteriovorus* recombinant strains were cultured in flasks in *E. coli* BL21(DE3) Gm^R^ suspensions for 24 h with their respective inducers (Table S5). The lysed co‐cultures were observed under fluorescence microscopy, detecting green fluorescence only in the strains with the P_JExD_ and the P_b_ promoters. In the other strains, green fluorescence was not detected.

**FIGURE 5:**
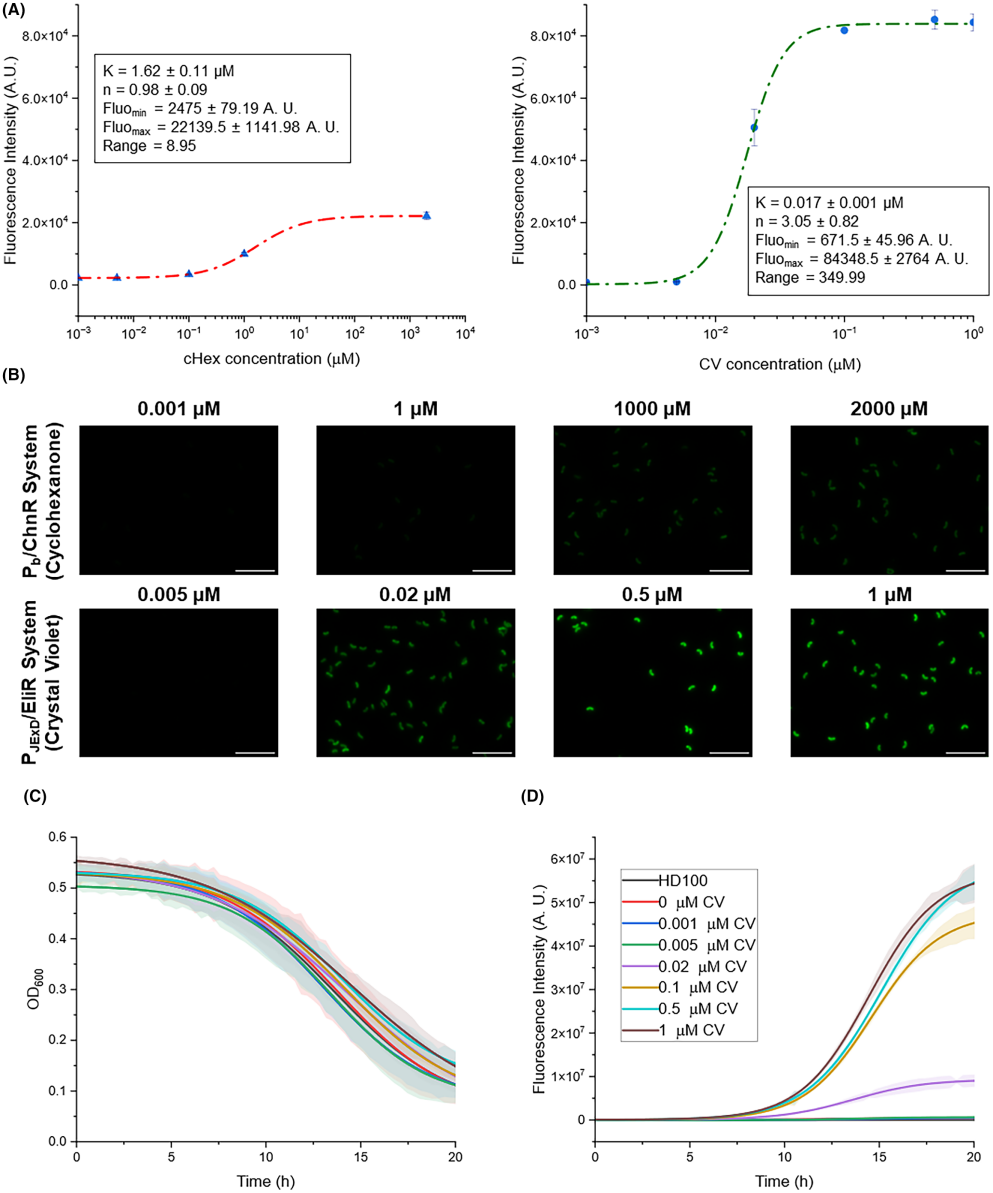
Characterization of inducible promoters. (A) Hill's curves of the P_b_/ChnB (left) and P_JExD_/EliR (right) expression systems. Fluorescence intensities of 24‐h predated co‐cultures with the indicated inductor's concentrations were measured by flow cytometry and fitted to Hill's curves using Origin 2022b (OriginLab, USA). Two biological replicates were analysed. (B) Fluorescence microscopy images. Scale bars represent 5 μm. (C) Absorbance curves of the prey, after blank subtraction (Hepes^+^). Points were fitted to a Richard curve using CuRveR. (D) Fluorescence intensity curves of each strain, after blank subtraction (HD100 strain). Points were fitted to a Richard curve using CuRveR. Error bars or filled areas in figure represent the SD between three biological replicates.

The lysed co‐cultures of the strains Bb08‐P_JExD_‐G and Bb08‐P_b_‐G were analysed by flow cytometry to measure the intensity of the fluorescence. As shown in Figure 5, both promoters are dose‐dependent, showing incremental levels of MsfGFP with the increasing concentrations of the respective inducer. Comparing the Hill's curves of both promoters (Figure 5A), the P_JExD_ presented a lower *K* parameter (or Hill constant), meaning that it needs a lower inducer concentration to occupy half of the available sites (Ang et al., 2013). Besides, the P_JExD_ promoter showed higher *msfGFP* expression levels with lower inducer concentration (the maximum was reached with 0.5 μM CV), whereas the P_b_ promoter needed 2 mM cHex to reach its maximal expression. Consequently, the P_JExD_ promoter showed a higher expression range, 350‐fold (Figure 5A). When both strains were analysed in the absence of the inducer, the population peaks showed a shift compared to the wild‐type strain (Figure S8). Despite the higher displacement of the P_b_ promoter harbouring strain, the fluorescence was not sufficient to be detected by microscopy (Figure 5B).

Considering the excellent behaviour of the P_JExD_ promoter harbouring strain, its absorbance and fluorescence were followed for 20 h using the wild‐type strain as control (Figure 5C,D). Co‐cultures were inoculated with the same initial predator cells (7.28 ± 0.03 Log PFUs mL^−1^, Table S9), using *E. coli* BL21(DE3) Gm^R^ at OD_600_ 1 in Hepes^+^ as prey. In Figure 5C, it can be seen that all the co‐cultures showed the same trend in terms of absorbance decrease (*r*_max_ = −3.79 ± 0.26 × 10^−2^ h^−1^, s = 13.84 ± 0.51, Table S9), meaning that the predation was not affected by the presence of the inducer nor the expression of the *msfGFP*. The fluorescence intensity, dependent on the CV concentration, increased with the growth of *B. bacteriovorus*, reaching saturation with the concentrations 0.5 and 1 μM (5.79 ± 0.15 × 10^7^ A. U., Table S9). In addition, the predated co‐cultures were analysed by flow cytometry to ensure that the different intensities were not due to different predator concentrations. Co‐cultures with a CV concentration higher than 0.005 μM had the same concentration of events at 24 h (from 9.13 ± 0.03 to 9.18 ± 0.02 Log events mL^−1^, Table S9). Therefore, the difference between intensities was due to the concentration of CV. All these results make this promoter suitable to control gene expression in *B. bacteriovorus* HD100.

## DISCUSSION

Great efforts have been recently made to enlighten the particular predatory lifestyle of *B. bacteriovorus* HD100, including the characterization of the predation at a nanometre‐scale (Kaplan et al., 2023) or the mechanisms behind the DNA replication dynamics (Kaljević et al., 2021, 2023). In addition, there has been a growing emphasis in understanding and developing its potential applications, including its use as a lytic agent for pathogen control in diverse fields such as aquaculture or agriculture, as well as its medical use in wound healing and as a probiotic (Cao et al., 2018; Dwidar et al., 2012; Liu et al., 2023; Sason et al., 2022). Besides, the powerful hydrolytic arsenal encoded in its genome is a valuable source for biotechnological industries.

The transformation of *B. bacteriovorus* HD100 into a microbial chassis highlights the necessity for genetic tools to streamline its “domestication.” The unique replication process of the predator in the bdelloplast, involving the formation of a filament that septates into multiple daughter cells, requires precise DNA partitioning (Kaljević et al., 2023). Consequently, the use of plasmids without partitioning signals can lead to heterogenous populations. In this work, we have demonstrated that *Bdellovibrio* mutants bearing replicative plasmids lead to heterogeneous populations even with selective pressure (Figures S5 and S6), making this approach unsuitable for certain purposes demanding a high degree of genetic and phenotypic consistency, such as large‐scale industrial processes.

The problem of heterogenous populations can be circumvented through chromosomal integration. In previous works, complete plasmids have been integrated into *B. bacteriovorus*' chromosome leading to merodiploids (Makowski et al., 2019; Martínez et al., 2016; Medina et al., 2008). However, these merodiploids must keep the selective pressure, that is, the antibiotic, to maintain the insertion. Otherwise, a new cross‐over event would lead to the plasmid excision and, consequently, to a heterogenous population. The chromosomal integration transposon Tn7‐mediated, presented in this work, is a robust method to easily integrate a single copy of a gene at a specific location of the chromosome, the *att*Tn7 site. Insertions of Tn7 transposons have been reported in bacteria such as *E. coli*, *P. putida*, *P. aeruginosa* or *B. subtilis* (Bordi et al., 2008; Choi & Schweizer, 2006; Lambertsen et al., 2004). Herein, we have demonstrated that transposon Tn7 integrates a single copy of the mobile element, which is flanked by Tn7L and Tn7R, downstream of the *glmS* gene in *B. bacteriovorus*. Although the Tn7 insertion in the *att*Tn7 site interrupts the ORF Bd3423, this gene has been predicted to be a hypothetical protein that cannot be identified using either BLAST or profile hidden Markov models (profile‐HMMs). Besides, we could not detect this protein in proteomic experiments carried out in our group (unpublished data). Therefore, we assume that this ORF is not translated in our experimental conditions or it is not an essential protein. In addition, the predation capabilities seem not to be affected by the Tn7 insertion (Figure 4B).

Although the transformation efficiencies achieved with *B. bacteriovorus* HD100 (10^−6^ transformant cells per initial recipient cells, Table 1) are low compared to those obtained in model organisms such as *E. coli* (Peters et al., 2019), all the strains that grew under Gm selection had the Tn7 insertion downstream the *glmS*. Furthermore, we have corroborated that the Tn7 insertion is stable and kept in the chromosome for ten generations without selective pressure. Therefore, the transposon Tn7‐mediated chromosomal integration is a robust method to obtain unique chromosomal insertion recombinants of *B. bacteriovorus* HD100 easily. The combination of this integration method with GS allows for the rapid construction of stable recombinant strains, taking advantage of the one‐pot hierarchical assembly of GS (Blázquez et al., 2023).

The availability of a battery of synthetic promoters for controlling the gene expression in *B. bacteriovorus* is a key challenge in the context of a microbial chassis, as there are instances where it is desirable to uncouple the expression of a specific gene from the growth phase. In this work, we have characterized in *B. bacteriovorus* HD100 six synthetic promoters designed for model organisms such as *E. coli* or *P. putida*, where they have been described as constitutive. In *B. bacteriovorus* HD100, these promoters showed a constitutive behaviour, and none of them represented a significant burden for the growth of the predator. The expression of reporter genes driven by these promoters resulted in a good approach to make *B. bacteriovorus* traceable during its growth. However, the expression levels sometimes differ from the data reported for other organisms. While promoters P_J102_, P_BG51_ and P_BG42_ showed similar behaviour in the predator as in the strains they were designed for, substantial differences were observed with P_J116_, P_J106_ and P_BG37_. The P_J116_, described as a low‐strength promoter in *E. coli*, showed medium strength in *B. bacteriovorus*. On the contrary, P_J106_, described as a medium‐strength promoter, showed very low strength in *B. bacteriovorus*, making it slightly different from the wild type (Figure 4A). Finally, P_BG37_ is described as medium‐low strength in *P. putida* and showed high expression in *B. bacteriovorus*.

The promoters from the “Anderson collection” have been characterized in other bacteria, such as *Chromobacterium violaceum*, *Actinobacillus succinogenes*, *Gluconacetobacter xylinus* or *Gluconacetobacter hansenii*, among others (Damalas et al., 2020; Liow et al., 2019; Teh et al., 2019), also showing different expression patterns even in species from the same genus. Besides, the promoters from the “Zobel collection,” developed for *P*. putida, showed a different profile in *E. coli* (Zobel et al., 2015). Generally, the variability in transcription levels among species is explained by differences in the transcription machinery. Although the regions involved in the promoter recognition RpoD of *E. coli* and *B. bacteriovorus* are similar (Figure S4), these small changes could influence the transcription levels.

Constitutive promoters are valuable tools when a constant gene expression is required. However, in certain scenarios, it could be required to control gene expression with an environmental cue. Previously, gene expression has been controlled in *B. bacteriovorus* by riboswitches, which consist of RNA regulatory elements that control the translation of the coding sequence of a mRNA through conformational changes caused by presence/absence of a ligand (aptamer) (Mandal & Breaker, 2004). Nonetheless, the Theo‐F riboswitch did not inhibit completely the translation (Dwidar & Yokobayashi, 2017). This is a common problem in model organisms, such as *E. coli*, that can be circumvented by combining different mechanisms to control the expression of genes, creating, for instance, repressive circuits (Chen et al., 2018). This is an example of the benefits of having gene expression regulatory elements, such as inducible/repressible promoters, in *B. bacteriovorus*. These elements would allow the development of fully tuned synthetic regulatory networks.

The carbohydrate promoters/regulator systems used in this work, P_BAD_/AraC and P_rhaBAD_/RhaSR, have been widely used to control gene expression in model and non‐model organism showing dose‐dependent induction and dynamic ranges higher than 100‐fold for the rhamnose‐induced promoter (Bi et al., 2013; Jeske & Altenbuchner, 2010; Loessner et al., 2007; Sukchawalit et al., 1999; Sydow et al., 2017). However, no *msfGFP* expression activation was observed under the control of these expression systems in *B. bacteriovorus* HD100. This behaviour can be explained by the bacterium's predatory lifestyle, which mainly feeds on amino acids (Herencias, Salgado‐Briegas, Prieto, & Nogales, 2020), without any transport systems described for these carbohydrates (Rendulic et al., 2004).

The P_m_/XylS promoter/regulator system derived from the *P. putida* TOL plasmid is induced by many benzoate‐derived compounds. Although the transport of these molecules is presumably passive (Lambert & Stratford, 1999), *msfGFP* expression could not be detected in *B. bacteriovorus* HD100 with increasing concentrations of 3 MB during co‐cultures. This absence of expression can be due to multiple factors: (1) 3 MB is not crossing the membrane efficiently; (2) the concentration of the XylS regulator, whose expression is driven by the native Ps2 promoter (Gawin et al., 2017), is not enough to trigger the transcription from the P_m_ promoter; and (3) XylS cannot recruit the RNA polymerase of *B. bacteriovorus* HD100. Further research would be needed to elucidate the answer.

In contrast, two inducible promoters were found to activate the expression of the *msfGFP* in the presence of their respective inducers. Although a light *msfGFP* induction was detected with the P_b_/ChnR promoter/regulator system in the absence of its inducer, this induction has also been detected in other hosts, such as *E. coli* and *P. fluorescens* (Steigedal & Valla, 2008). In contrast, the P_JExD_/EliR promoter/regulator system presented tight regulation with negligible induction without its inducer. This system showed higher *msfGFP* expression with less concentration of the inducer, having a better induction range than the P_b_/ChnB system, in line with the results obtained in model microorganisms such as *E. coli* or *P. putida* (Ruegg et al., 2018). Altogether, the P_JExD_/EliR is an excellent candidate for use in *B. bacteriovorus* HD100 when tight regulation is needed. In this context, it is worth to mention that the genetic tools developed in our study establish the groundwork for development this predatory bacterium as a model microbial chassis, allowing for example genome editing through CRISPR/Cas9 (Kuscu et al., 2014) or other systems that requires tight regulation of gene expression associated with constitutive gene expression.

## CONCLUSIONS

Through this research, we have expanded the repertoire of genetic tools, facilitating the manipulation of this intriguing predatory bacterium. The battery of promoters characterized in *B. bacteriovorus* HD100 allows for fine‐tuning and controlled heterologous gene expression either constitutively or in an inducible manner. These promoters, along with the chromosomal insertion transposon Tn7 mediated, ease the generation of recombinant strains. This not only aids in the study of protein functions but also paves the way to control *B. bacteriovorus* HD100 in two‐membered bacterial systems.

## AUTHOR CONTRIBUTIONS

**Sergio Salgado:** Conceptualization; data curation; formal analysis; visualization; writing – original draft; methodology; investigation; writing – review and editing; validation. **Natalia Hernández‐Herreros:** Methodology; investigation; formal analysis; writing – original draft; writing – review and editing. **M. Auxiliadora Prieto:** Conceptualization; supervision; funding acquisition; validation; writing – review and editing; project administration; investigation.

## CONFLICT OF INTEREST STATEMENT

The authors declare no conflict of interest.

## Supporting information


Appendix S1.

## Data Availability

The data that supports the findings of this study are available in the supplementary material of this article.
