# Supplementary material for: Controlling the expression of heterologous genes in Bdellovibrio bacteriovorus using synthetic biology strategies
Source: Microb Biotechnol. 2024 Jun 27;17(6):e14517. doi: 10.1111/1751-7915.14517 (PMC11209729; doi:10.1111/1751-7915.14517)
Supplement: Supplementary file 1 — Appendix S1. [file MBT2-17-e14517-s001.pdf]

Supplementary material for the research article “Controlling the Expression of Heterologous Genes in *Bdellovibrio bacteriovorus* using Synthetic Biology Strategies”

Sergio Salgado[1,2], Natalia Hernández-Herreros[1,2], and M. Auxiliadora Prieto[1,2*]

[1]Interdisciplinary Platform for Sustainable Plastics towards a Circular Economy-Spanish National Research Council (SusPlast-CSIC), Madrid, Spain.

[2]Polymer Biotechnology Group, Department of Microbial and Plant Biotechnology, Margarita Salas Center for Biological Research (CIB-CSIC), Madrid, Spain.

**Corresponding author.*

Supplementary Materials and Methods

Molecular biology reagents

Plasmid DNA minipreps were made using the High Pure Plasmid Isolation Kit (Roche, Switzerland) according to the manufacturer's instructions. DNA agarose gel bands, PCR products, and digestion products were purified with the QIAquick Gel Extraction Kit (QIAGEN, Germany). DNA concentrations were measured using a NanoDrop 2000 Spectrophotometer (ThermoFisher Scientific, USA). The GS restriction enzymes, BpiI and BsaI, were acquired from ThermoFisher Scientific. Phusion High-Fidelity DNA polymerase, T4 DNA ligase, and all other restriction enzymes were purchased from New England Biolabs (USA). For screening purposes, NZYTaq II 2x Green Master Mix (NZYTech, Portugal) was employed. Chemically competent *E. coli* cells were prepared as described elsewhere (Inoue et al., 1990), with modifications: an *E. coli* overnight preculture was utilized to inoculate SOB medium (2 % Bacto-tryptone, 0.5 % Yeast extract, 10 mM NaCl, 2.5 mM KCl, 10 mM $MgCl_2$, 10 mM $MgSO_4$) to achieve an OD_{600} 0.05. The inoculum was incubated for 48 h at 200 rpm and 18 °C. After reaching OD_{600} ~ 0.6, the culture was cooled on ice for 10 min. Subsequently, 100 mL were centrifuged at 3000 rpm and 4 °C for 10 min, followed by resuspension in 40 mL of precooled TB buffer (10 mM Hepes, 15 mM $CaCl_2$, 250 mM KCl, 55 mM $MnCl_2$, pH 6.7) and incubated for another 10 min on ice. The suspension was then subjected to a second round of centrifugation at 3000 rpm and 4 °C for 10 min and resuspended in 10 mL of precooled TB buffer. DMSO was incrementally added to the sample to achieve a final concentration of 7 %, and the mixture was further incubated for 10 min on ice. Cell suspension was divided into 100 µL aliquots, immediately frozen in liquid nitrogen, and stored at -80 °C.

Supplementary Tables

Table S1. List of strains used throughout this work.

Strain	Genotype	Reference
E. coli DH5α	Cloning host; F- λ^- *endAI hsdRJ7* (r_K^-, m_K^+) *supE44 thi-1 recA1 gyrA96 relA1 deoR* Δ(*lacZYA-argF*)U169 φ80d*lacZ*ΔM15	(Grant et al., 1990)
E. coli DH5α λ*pir*$^+$	Cloning host for R6K *ori* plasmids; λpir phage lysogen of *E. coli* DH5α	(Platt et al., 2000)
E. coli DH10B	Cloning host; F^- *araD139* Δ(*ara*, *leu*)7697 Δ*lacX74 galU galK rpsL deoR* 80d*lacZ*ΔM15 *endA1 nupG recA1 mcrA* Δ(*mrr hsdRMS mcrBC*)	(Grant et al., 1990)
E. coli CC118 λ*pir*$^+$	Cloning host for R6K *ori* plasmids; Rf^R Sp^R; Δ(*ara-leu*) *araD*Δ*lacX74 galE galK phoA20 thi-1 rpsE rpoB argE*(Am) *recA1*; λ*pir*$^+$ phage lysogen of *E. coli* CC118	(Herrero et al., 1990)
E. coli HB101	Host for pRK600 plasmid, used as mating helper strain; F^- λ^- *mcrB mrr hsdS20*(r_B^- m_B^-) *recA13 leuB6 ara-14 proA2 lacY1 galK2 xyl-5 mtl-1 rpsL20(Sm^R) gln V44*	(Boyer and Roulland-dussoix, 1969)
E. coli BL21(DE3)	F^- *ompT gal dcm lon hsdS*$_B$(r_B^- m_B^-) λ(DE3 [*lacI lacUV5-T7p07 ind1 sam7 nin5*]) [*malB*$^+$]$_{K-12}$(λ^S)	(Studier and Moffatt, 1986)
E. coli BL21(DE3) Gm^R	Prey strain; Gm^R, *E. coli* BL21(DE3) strain with genomic insertion of pTn7-19[g2]-ncTU	This work
P. putida KT2440	TOL plasmid-cured, spontaneous restriction deficient derivative of *P. putida* mt-2	(Bagdasarian et al., 1981)
P. putida KT2440 Gm^R	Prey strain; Gm^R, *P. putida* KT2440 strain with genomic insertion of pTn7-19[g2]-ncTU	This work
B. bacteriovorus HD100	Wild-type strain (DSMZ 50701)	(Stolp and Starr, 1963)
Bb08-ncTU	Gm^R, *B. bacteriovorus* HD100 strain with genomic insertion of pTn7-19[g2]-ncTU	This work
Bb08-P_0-R	Gm^R, *B. bacteriovorus* HD100 strain with genomic insertion of pTn7-19[g2]-P_0-R	This work
Bb08-P_{J116}-R	Gm^R, *B. bacteriovorus* HD100 strain with genomic insertion of pTn7-19[g2]-P_{J116}-R	This work
Bb08-P_{J102}-R	Gm^R, *B. bacteriovorus* HD100 strain with genomic insertion of pTn7-19[g2]-P_{J102}-R	This work

Strain	Genotype	Reference
Bb08-P_{J106}-R	Gm^R, *B. bacteriovorus* HD100 strain with genomic insertion of pTn7-19[g2]-P_{J106}-R	This work
Bb08-P_{BG37}-R	Gm^R, *B. bacteriovorus* HD100 strain with genomic insertion of pTn7-19[g2]-P_{BG37}-R	This work
Bb08-P_{BG42}-R	Gm^R, *B. bacteriovorus* HD100 strain with genomic insertion of pTn7-19[g2]-P_{BG42}-R	This work
Bb08-P_{BG25}-R	Gm^R, *B. bacteriovorus* HD100 strain with genomic insertion of pTn7-19[g2]-P_{BG25}-R	This work
Bb08-P_{BAD}-G	Gm^R, *B. bacteriovorus* HD100 strain with genomic insertion of pTn7-19[gE]-P_{BAD}-G	This work
Bb08-P_m-G	Gm^R, *B. bacteriovorus* HD100 strain with genomic insertion of pTn7-19[gE]-P_m-G	This work
Bb08-P_{JExD}-G	Gm^R, *B. bacteriovorus* HD100 strain with genomic insertion of pTn7-19[gE]-P_{JExD}-G	This work
Bb08-P_{rhaBAD}-G	Gm^R, *B. bacteriovorus* HD100 strain with genomic insertion of pTn7-19[gE]-P_{rhaBAD}-G	This work
Bb08-P_b-G	Gm^R, *B. bacteriovorus* HD100 strain with genomic insertion of pTn7-19[gE]-P_b-G	This work

Gm^R: gentamicin resistant

Table S2. List of plasmids used in this work.

Plasmid name	Description	Reference[a]
pRK600	Helper plasmid used for conjugation; Cm^R, *ori* ColE1, *mob*$^+$ *tra*$^+$ of RK2	(Kessler et al., 1992)
pTnS-1	Plasmid for Tn7-transposon insertion; Ap^R, *ori* R6K, *TnSABC+D* operon	(Choi et al., 2005)
**For Modular Cloning adaption**		
pSEVA2319[g2]	Km^R, *ori* pBBR1; Golden Standard host vector, Level 1 Position 2, used as PCR template for subcloning	(Blázquez et al., 2023)
pSEVA6319[gE]	Gm^R, *ori* pBBR1; Golden Standard host vector, Level 2 Position E, used as PCR template for subcloning	(Blázquez et al., 2023)
pSEVA251	Km^R, *ori* RSF1010; used for Golden Standard adaption	(Martínez-García et al., 2023)
pSEVA251-dom	Km^R, *ori* RSF1010; pSEVA251 plasmid domesticated for BsaI and BpiI enzymes	This work
pSEVA2519[g2]	Km^R, *ori* RSF1010; Golden Standard host vector, Level 1 Position 2 with *lacZα*	This work (PP068168)
pSEVA2519[gE]	Km^R, *ori* RSF1010; Golden Standard host vector, Level 2 Position E with *lacZα*	This work (PP068171)
pSEVA651	Gm^R, *ori* RSF1010 used for Golden Standard adaption	(Martínez-García et al., 2023)
pSEVA651-dom	Gm^R, *ori* RSF1010; pSEVA251 plasmid domesticated for BsaI and BpiI enzymes	This work
pSEVA6519[g2]	Gm^R, *ori* RSF1010; Golden Standard host vector, Level 1 Position 2 with *lacZα*	This work (PP068173)
pSEVA6519[gE]	Gm^R, *ori* RSF1010; Golden Standard host vector, Level 2 Position E with *lacZα*	This work (PP068182)
pTn7-M	Km^R Gm^R, *ori* R6K, *Tn7L* and *Tn7R* extremes, *lacZα,* standard multiple cloning site; used for Golden Standard adaption	(Zobel et al., 2015)
pTn7-19[g2]	Km^R Gm^R, *ori* R6K, *Tn7L* and *Tn7R* extremes, pTn7-M plasmid adapted for Golden Standard Level 1 position 2 with *lacZα*	This work (PP068183)
pTn7-19[gE]	Km^R Gm^R, *ori* R6K, *Tn7L* and *Tn7R* extremes, pTn7-M plasmid adapted for Golden Standard Level 2 position E with *lacZα*	This work (PP068192)

Plasmid name	Description	Reference[a]
**Level 0 plasmids**		
pL0-PUSCT	Sp/Sm^R, *ori* pUC/ColE1; Golden Gate Level 0 acceptor position AI with *lacZα*	(Weber et al., 2011)
pL0-ncTU-AI	Sp/Sm^R, *ori* pUC/ColE1; pL0-PUSCT derivative containing a non-coding sequence.	This work (PP068162)
pSEVA6331	Gm^R, *ori* pBBR1; vector encoding the cyclohexanone-inducible ChnR/P_b system	(Benedetti et al., 2016; Martínez-García et al., 2023)
pL0-ChnR-AI	Sp/Sm^R, *ori* pUC/ColE1; pL0-PUSCT derivative containing *chrR* TU.	This work (PP068161)
pL0-SC	Sp/Sm^R, *ori* pUC/ColE1; Golden Gate Level 0 acceptor position DG with *lacZα*	(Weber et al., 2011)
C0080_CD	Amp^R, *ori* pUC; CIDAR vector containing the AraC regulator	(Iverson et al., 2016)
pL0-AraC-DG	Sp/Sm^R, *ori* pUC/ColE1; pL0-SC derivative, *araC* CDS part	This work (PP068160)
pL0-P	Sp/Sm^R, *ori* pUC/ColE1; Golden Gate Level 0 acceptor position AB with *lacZα*	(Weber et al., 2011)
pL0-P_0-AB	Ap^R, *ori* pUC; pSEVA182 derivative plasmid with a non-functional promoter sequence (Linker 1_AB) at position AB	(Blázquez et al., 2023)
pL0-P_{J116}-AB	Ap^R, *ori* pUC; pSEVA182 derivative plasmid with P_{J23116} promoter at position AB	(Blázquez et al., 2023)
pL0-P_{J102}-AB	Ap^R, *ori* pUC; pSEVA182 derivative plasmid with P_{J23102} promoter at position AB	(Blázquez et al., 2023)
pL0-P_{J106}-AB	Ap^R, *ori* pUC; pSEVA182 derivative plasmid with P_{J23106} promoter at position AB	(Blázquez et al., 2023)
pL0-P_{BG37}-AB	Ap^R, *ori* pUC; pSEVA182 derivative plasmid with P_{BG37} promoter at position AB	(Blázquez et al., 2023)
pL0-P_{BG42}-AB	Ap^R, *ori* pUC; pSEVA182 derivative plasmid with P_{BG42} promoter at position AB	(Blázquez et al., 2023)
pL0-P_{BG25}-AB	Ap^R, *ori* pUC; pSEVA182 derivative plasmid with P_{BG25} promoter at position AB	(Blázquez et al., 2023)
pL0-P_{BAD}-AB	Ap^R, *ori* pUC; pSEVA182 derivative plasmid with P_{BAD} promoter at position AB	(Blázquez et al., 2023)
pL0-P_m-AB	Ap^R, *ori* pUC; pSEVA182 derivative plasmid with P_m promoter at position AB	(Blázquez et al., 2023)

Plasmid name	Description	Reference[a]
pL0-P_{JExD}-AB	Ap^R, *ori* pUC; pSEVA182 derivative plasmid with P_{JExD} promoter at position AB	(Blázquez et al., 2023)
pL0-P_{rhaBAD}-AB	Ap^R, *ori* pUC; pSEVA182 derivative plasmid with P_{rhaBAD} promoter at position AB	(Blázquez et al., 2023)
pL0-P_b-AB	Sp/Sm^R, *ori* pUC/ColE1; pL0-P derivative plasmid with P_b promoter at position AB	This work (PP068163)
pL0-BCD2-BD	ApR, *ori* pUC; pSEVA182 derivative plasmid with BCD2 RBS at position BD	(Blázquez et al., 2023)
pL0-mRFP1-DG	ApR, *ori* pUC; pSEVA182 derivative plasmid with *mRFP1* CDS at position DG	(Blázquez et al., 2023)
pL0-msfGFP-DG	Sp/Sm^R, *ori* pUC/ColE1; pL0-SC derivative plasmid with *msfGFP* CDS at position DG	(Blázquez et al., 2023)
pL0-rnpB_T1-GI	Sp/Sm^R, *ori* pUC/ColE1; pL0-T derivative plasmid with rnpB_T1 terminator at position GI	(Blázquez et al., 2023)
**Level 1 plasmids**		
pSEVA2319EliR[g2]	Km^R, *ori* pBBR1; Golden Standard host vector, Level 1 position 2, including EliR in position 1.	(Blázquez et al., 2023)
pSEVA2319[g1]	Km^R, *ori* pBBR1; Golden Standard host vector, Level 1 Position 1 with *lacZα*	(Blázquez et al., 2023)
pSEVA2319[g2]	Km^R, *ori* pBBR1; Golden Standard host vector, Level 1 Position 2 with *lacZα*	(Blázquez et al., 2023)
pSEVA2819[g3]	Km^R, *ori* pUC; Golden Standard host vector, Level 1 Position 3 with *lacZα*	(Blázquez et al., 2023)
pSEVA2319[g3]	Km^R, *ori* pBBR1; Golden Standard host vector, Level 1 Position 3 with *lacZα*	(Blázquez et al., 2023)
pSEVA181-L_23	Amp^R, *ori* pUC; Golden Standard linker with 23 fusion sites	(Blázquez et al., 2023)
pSEVA181-L_34	Amp^R, *ori* pUC; Golden Standard linker with 34 fusion sites	(Blázquez et al., 2023)
pSEVA181-L_45	Amp^R, *ori* pUC; Golden Standard linker with 45 fusion sites	(Blázquez et al., 2023)
pSEVA181-L_56	Amp^R, *ori* pUC; Golden Standard linker with 56 fusion sites	(Blázquez et al., 2023)
pL1F-Dummy_6	Amp^R, *oris* pUC and RK2; Golden Gate Level 1 vector containing linker with 67 fusion sites.	(Weber et al., 2011)
pSEVA6519[g2]-ncTU	Gm^R, *ori* RSF1010; Level 1 pSEVA6519[g2] vector containing non-coding sequence	This work (PP068174)

Plasmid name	Description	Reference[a]
pSEVA6519[g2]-P_0-R	Gm^R, *ori* RSF1010; Level 1 pSEVA6519[g2] vector containing P_0-BCD2-*mRFP1*-rnpB_T1	This work (PP068175)
pSEVA6519[g2]-P_{J102}-R	Gm^R, *ori* RSF1010; Level 1 pSEVA6519[g2] vector containing P_{J23102}-BCD2-*mRFP1*-rnpB_T1	This work (PP068179)
pSEVA6519[g2]-P_{J106}-R	Gm^R, *ori* RSF1010; Level 1 pSEVA6519[g2] vector containing P_{J23106}-BCD2-*mRFP1*-rnpB_T1	This work (PP068180)
pSEVA6519[g2]-P_{J116}-R	Gm^R, *ori* RSF1010; Level 1 pSEVA6519[g2] vector containing P_{J23116}-BCD2-*mRFP1*-rnpB_T1	This work (PP068181)
pSEVA6519[g2]-P_{BG25}-R	Gm^R, *ori* RSF1010; Level 1 pSEVA6519[g2] vector containing P_{BG25}-BCD2-*mRFP1*-rnpB_T1	This work (PP068176)
pSEVA6519[g2]-P_{BG37}-R	Gm^R, *ori* RSF1010; Level 1 pSEVA6519[g2] vector containing P_{BG37}-BCD2-*mRFP1*-rnpB_T1	This work (PP068177)
pSEVA6519[g2]-P_{BG42}-R	Gm^R, *ori* RSF1010; Level 1 pSEVA6519[g2] vector containing P_{BG42}-BCD2-*mRFP1*-rnpB_T1	This work (PP068178)
pTn7-19[g2]-ncTU	Km^R Gm^R, *ori* R6K; Level 1 pTn7-19[g2] vector containing non-coding sequence	This work (PP068184)
pTn7-19[g2]-P_0-R	Km^R Gm^R, *ori* R6K; Level 1 pTn7-19[g2] vector containing P_0-BCD2-*mRFP1*-rnpB_T1	This work (PP068185)
pTn7-19[g2]-P_{J102}-R	Km^R Gm^R, *ori* R6K; Level 1 pTn7-19[g2] vector containing P_{J23102}-BCD2-*mRFP1*-rnpB_T1	This work (PP068189)
pTn7-19[g2]-P_{J106}-R	Km^R Gm^R, *ori* R6K; Level 1 pTn7-19[g2] vector containing P_{J23106}-BCD2-*mRFP1*-rnpB_T1	This work (PP068190)
pTn7-19[g2]-P_{J116}-R	Km^R Gm^R, *ori* R6K; Level 1 pTn7-19[g2] vector containing P_{J23116}-BCD2-*mRFP1*-rnpB_T1	This work (PP068191)
pTn7-19[g2]-P_{BG25}-R	Km^R Gm^R, *ori* R6K; Level 1 pTn7-19[g2] vector containing P_{BG25}-BCD2-*mRFP1*-rnpB_T1	This work (PP068186)
pTn7-19[g2]-P_{BG37}-R	Km^R Gm^R, *ori* R6K; Level 1 pTn7-19[g2] vector containing P_{BG37}-BCD2-*mRFP1*-rnpB_T1	This work (PP068187)
pTn7-19[g2]-P_{BG42}-R	Km^R Gm^R, *ori* R6K; Level 1 pTn7-19[g2] vector containing P_{BG42}-BCD2-*mRFP1*-rnpB_T1	This work (PP068188)
pSEVA2819[g1R]-XylS	Km^R, *ori* pUC; Level 1 pSEVA2819[g2] vector containing XylS	(Blázquez et al., 2023)
pSEVA2319[g1]-RhaSR	Km^R, *ori* pBBR1; Level 1 pSEVA2319[g2] vector containing RhaSR	(Blázquez et al., 2023)
pSEVA2819[g1R]-EliR	Km^R, *ori* pUC; Level 1 pSEVA2819[g2] vector containing EliR	(Blázquez et al., 2023)
pSEVA2319[g1]-ChnR	Km^R, *ori* pBBR1; Level 1 pSEVA2319[g2] vector containing ChnR	This work (PP068165)

Plasmid name	Description	Reference[a]
pSEVA2319[g1]-P_{J106}-AraC	Km^R, *ori* pBBR1; Level 1 pSEVA2319[g2] vector containing P_{J23106}-BCD2-*araC*-rnpB_T1	This work (PP068166)
pSEVA2519[g2]-P_m-G	Km^R, *ori* RSF1010; Level 1 pSEVA2519[g2] vector containing P_m-BCD2-*msfGFP*-rnpB_T1	This work (PP068169)
pSEVA2519[g2]-P_{rhaBAD}-G	Km^R, *ori* RSF1010; Level 1 pSEVA2519[g2] vector containing P_{rhaBAD}-BCD2-*msfGFP*-rnpB_T1	This work (PP068170)
pSEVA2319EliR[g2]-P_{JExD}-G	Km^R, *ori* pBBR1; Level 1 pSEVA2319EliR[g2] vector containing P_{JExD}-BCD2-*msfGFP*-rnpB_T1	This work (PP068164)
pSEVA2319[g2]-P_b-G	Km^R, *ori* pBBR1; Level 1 pSEVA2319[g2] vector containing P_b-BCD2-*msfGFP*-rnpB_T1	This work (PP068167)
pSEVA2819[g3]-P_{BAD}-G	Km^R, *ori* pUC; Level 1 pSEVA2819[g2] vector containing P_{BAD}-BCD2-*msfGFP*-rnpB_T1	This work (PP068172)
**Level 2 plasmids**		
pTn7-19[gE]-P_m-G	Km^R Gm^R, *ori* R6K; Level 2 pTn7-19[gE] vector containing XylS-P_m-BCD2-*msfGFP*-rnpB_T1	This work (PP068196)
pTn7-19[gE]-P_{rhaBAD}-G	Km^R Gm^R, *ori* R6K; Level 2 pTn7-19[gE] vector containing RhaSR-P_{rhaBAD}-BCD2-*msfGFP*-rnpB_T1	This work (PP068197)
pTn7-19[gE]-P_{JExD}-G	Km^R Gm^R, *ori* R6K; Level 2 pTn7-19[gE] vector containing EliR-P_{JExD}-BCD2-*msfGFP*-rnpB_T1	This work (PP068195)
pTn7-19[gE]-P_b-G	Km^R Gm^R, *ori* R6K; Level 2 pTn7-19[gE] vector containing ChnR-P_b-BCD2-*msfGFP*-rnpB_T1	This work (PP068194)
pTn7-19[gE]-P_{BAD}-G	Km^R Gm^R, *ori* R6K; Level 2 pTn7-19[gE] vector containing AraC-P_{BAD}-BCD2-*msfGFP*-rnpB_T1	This work (PP068193)

Gm^R, gentamicin resistance; Km^R, kanamycin resistance; Ap^R: ampicillin resistance; Sp^R: spectinomycin resistance; Sm^R: streptomycin resistance; Tet^R: tetracycline resistance; Cm^R: chloramphenicol resistance.

[a] The GenBank accession numbers of the constructed plasmid are included within parentheses.

Table S3. Oligonucleotides used in this work.

Oligonucleotide	Sequence	Purpose
SS61	GTGGGCGGGTCCTCGGCGGGCAGGTAG	Domestication of pSEVA plasmids
SS62	CTACCTGCCCGCCGAGGACCCGCCCAC	Domestication of pSEVA plasmids
SS63	GAAAGGCTTGTCCTCGCGGAAC	Domestication of pSEVA plasmids
SS64	GTTCCGCGAGGACAAGCCTTTC	Domestication of pSEVA plasmids
RK81	GGAAGAGCGCCCAATACG	GS sequencing
RK82	AAAGTGCCACCTGACGTCTA	GS sequencing
PS1	AGGGCGGCGGATTTGTCC	GS sequencing
PS2	GCGGCAACCGAGCGTTC	GS sequencing
pBG-Fwd	CAAGGTTCTGGACCAGTTGCG	GS sequencing
SS147	TGAACAACATCATGACCACCTG	PCR amplification of *B. bacteriovorus* Tn7 insertions
SS126	GTCAGATGGAAGGTGTTTTGTC	PCR amplification of *B. bacteriovorus* Tn7 insertions
SS204	GATGATCGGTTCAGCCATCTGC	Sequencing of *B. bacteriovorus* Tn7 insertions
SS205	GCAAGGTGATTTCTATCGGCAC	Sequencing of *B. bacteriovorus* Tn7 insertions
SS208	TTTGAAGACGTggagGCAACATACGCTGGACGCTACGTCTTCAAA	pL0-ncTU-AI construction
SS209	TTTGAAGACGTagcgTCCAGCGTATGTTGCCTCCACGTCTTCAAA	pL0-ncTU-AI construction
SS222	TTTGAAGACTGaagcTTAAGCTACTAAAGCGTAGTTTTCG	pL0-AraC-DG construction
MM382	TTTGAAGACGTggagGCAACTAAAAGAGATTGTTTGGATCA	pL0-P_b-AB construction
MM383	TTTGAAGACGTggagGCAACTAAAAGAGATTGTTTGGATCA	pL0-P_b-AB construction
MM384	TTTGAAGACACggagGTTTGACAGCTTATCATCGACTGC	pL0-ChnR-AI construction
MM385	TTTGAAGACACagcgTCTAGGGCGGCGGATTTGTC	pL0-ChnR-AI construction

Table S4. Synthetic constitutive promoters used in this study.

Promoter[a]	Strength[b]	Sequence[c]
J23116[1]	L[4]	TTGACAGCTAGCTCAGTCCTAGGGACTATGCTAGC
J23106[1]	M[4]	TTTACGGCTAGCTCAGTCCTAGGTATAGTGCTAGC
J23102[1]	H[4]	TTGACAGCTAGCTCAGTCCTAGGTACTGTGCTAGC
BG37[2]	L[5]/M-L[6]	GTGAATTGACATGTCAATTTTTATGTTGTATAATATAACTA
BG25[2]	M[5]/[6]	GCCCGTTGACATGACATGGTTTTGAGGGTATAATGTGGCGA
BG42[2]	H[5,6]	GCCCATTGACAAGGCTCTCGCGGCCAGGTATAATTGCACGA
P_0[3]	None	CCCCTGGCGCCCCTT

Abbreviations: Low (L); Medium (M); High (H)

[a] Source:

1. Anderson collection (http://parts.igem.org/Promoters/Catalog/Anderson).
2. Zobel collection (Zobel et al., 2015)
3. GS collection (Blázquez et al., 2023)

[b] Strength measured in:

4. *E. coli* (http://parts.igem.org/Promoters/Catalog/Anderson, (Chappell et al., 2013)).
5. *E. coli* (Zobel et al., 2015)
6. *P. putida* (Zobel et al., 2015)

[c] -35 and -10 boxes are underlined.

Table S5. Concentration ranges of inducers used for each strain.

Strain	Inducer	Concentration range
Bb08-P_{BAD}-G	L-Arabinose	0-100 mM
Bb08-P_{rhaBAD}-G	L-Rhamnose	0-100 mM
Bb08-P_{m}-G	3-Methylbenzoate (3MB)	0-10 mM
Bb08-P_{JExD}-G	Crystal violet (CV)	0-1 µM
Bb08-P_{b}-G	Cyclohexanone (cHex)	0-2 mM

Table S6. Constructed Golden Standard Level 0 parts

Name	Level	Primers for PCR / MoClo reaction	Template for PCR	Backbone	Position	Restriction enzyme†	5' Fusion site	3' Fusion site	Antibiotic Resistance	Origin of Replication
**pL0-ncTU-AI**	0	SS208-SS209	*	pL0-PUSCT	AI	BpiI	GGAG	CGCT	Sp/Sm	pMB1
**pL0-AraC-DG**	0	SS221-SS222	C0080_CD	pL0-SC	DG	BpiI	AATG	GCTT	Sp/Sm	pMB1
**pL0-P_b-AB**	0	MM382 – MM383	pSEVA6311	pL0-P	AB	BpiI	GGAG	TACT	Sp/Sm	pMB1
**pL0-ChnR-AI**	0	MM384 – MM385	pSEVA6311	pL0-PUSCT	AI	BpiI	GGAG	CGCT	Sp/Sm	pMB1

† Restriction enzyme used to make construct; Sp, streptomycin; Sm, spectinomycin.

* GS reaction was done with 100 ng of annealed primers instead of PCR product.

Table S7. Detailed Golden Standard Level 1 and Level 2 constructions

Name	Level	Component Plasmid(s)	Backbone	Position	Restriction Enzyme†	5' Fusion site	3' Fusion site	Antibiotic Resistance	Origin of Replication
**pSEVA6519[g2]-ncTU**	1	pL0-ncTU-AI	pSEVA6519[g2]	2	BsaI	GCAA	ACTA	Gm	RSF1010
**pSEVA6519[g2]-P_0**	1	pL0-P_0-AB<br>pL0-BCD2-BD<br>pL0-mRFP1-DG<br>pL0-rnpB_T1-GI	pSEVA6519[g2]	2	BsaI	GCAA	ACTA	Gm	RSF1010
**pSEVA6519[g2]-P_{J102}-R**	1	pL0-P_{J102}-AB<br>pL0-BCD2-BD<br>pL0-mRFP1-DG<br>pL0-rnpB_T1-GI	pSEVA6519[g2]	2	BsaI	GCAA	ACTA	Gm	RSF1010
**pSEVA6519[g2]-P_{J106}-R**	1	pL0-P_{J106}-AB<br>pL0-BCD2-BD<br>pL0-mRFP1-DG<br>pL0-rnpB_T1-GI	pSEVA6519[g2]	2	BsaI	GCAA	ACTA	Gm	RSF1010
**pSEVA6519[g2]-P_{J116}-R**	1	pL0-P_{J116}-AB<br>pL0-BCD2-BD<br>pL0-mRFP1-DG<br>pL0-rnpB_T1-GI	pSEVA6519[g2]	2	BsaI	GCAA	ACTA	Gm	RSF1010
**pSEVA6519[g2]-P_{BG37}-R**	1	pL0-P_{BG37}-AB<br>pL0-BCD2-BD<br>pL0-mRFP1-DG<br>pL0-rnpB_T1-GI	pSEVA6519[g2]	2	BsaI	GCAA	ACTA	Gm	RSF1010
**pSEVA6519[g2]-P_{BG42}-R**	1	pL0-P_{BG42}-AB<br>pL0-BCD2-BD<br>pL0-mRFP1-DG<br>pL0-rnpB_T1-GI	pSEVA6519[g2]	2	BsaI	GCAA	ACTA	Gm	RSF1010
**pSEVA6519[g2]-P_{BG25}-R**	1	pL0-P_{BG25}-AB<br>pL0-BCD2-BD<br>pL0-mRFP1-DG<br>pL0-rnpB_T1-GI	pSEVA6519[g2]	2	BsaI	GCAA	ACTA	Gm	RSF1010
**pTn7-19[g2]-ncTU**	1	pL0-ncTU-AI	pTn7-19[g2]	2	BsaI	GCAA	ACTA	Gm/Km	R6K

Name	Level	Component Plasmid(s)	Backbone	Position	Restriction Enzyme†	5' Fusion site	3' Fusion site	Antibiotic Resistance	Origin of Replication
**pTn7-19[g2]-P_0**	1	pL0-P_0-AB<br>pL0-BCD2-BD<br>pL0-mRFP1-DG<br>pL0-rnpB_T1-GI	pTn7-19[g2]	2	BsaI	GCAA	ACTA	Gm/Km	R6K
**pTn7-19[g2]-P_{J102}-R**	1	pL0-P_{J102}-AB<br>pL0-BCD2-BD<br>pL0-mRFP1-DG<br>pL0-rnpB_T1-GI	pTn7-19[g2]	2	BsaI	GCAA	ACTA	Gm/Km	R6K
**pTn7-19[g2]-P_{J106}-R**	1	pL0-P_{J106}-AB<br>pL0-BCD2-BD<br>pL0-mRFP1-DG<br>pL0-rnpB_T1-GI	pTn7-19[g2]	2	BsaI	GCAA	ACTA	Gm/Km	R6K
**pTn7-19[g2]-P_{J116}-R**	1	pL0-P_{J116}-AB<br>pL0-BCD2-BD<br>pL0-mRFP1-DG<br>pL0-rnpB_T1-GI	pTn7-19[g2]	2	BsaI	GCAA	ACTA	Gm/Km	R6K
**pTn7-19[g2]-P_{BG37}-R**	1	pL0-P_{BG37}-AB<br>pL0-BCD2-BD<br>pL0-mRFP1-DG<br>pL0-rnpB_T1-GI	pTn7-19[g2]	2	BsaI	GCAA	ACTA	Gm/Km	R6K
**pTn7-19[g2]-P_{BG42}-R**	1	pL0-P_{BG42}-AB<br>pL0-BCD2-BD<br>pL0-mRFP1-DG<br>pL0-rnpB_T1-GI	pTn7-19[g2]	2	BsaI	GCAA	ACTA	Gm/Km	R6K
**pTn7-19[g2]-P_{BG25}-R**	1	pL0-P_{BG25}-AB<br>pL0-BCD2-BD<br>pL0-mRFP1-DG<br>pL0-rnpB_T1-GI	pTn7-19[g2]	2	BsaI	GCAA	ACTA	Gm/Km	R6K
**pSEVA2319[g1]-P_{J106}-AraC**	1	pL0-P_{J106}-AB<br>pL0-BCD2-BD<br>pL0-AraC-DG<br>pL0-rnpB_T1-GI	pSEVA2319[g1]	1	BsaI	TGCC	GCAA	Km	pBBR1

Name	Level	Component Plasmid(s)	Backbone	Position	Restriction Enzyme†	5' Fusion site	3' Fusion site	Antibiotic Resistance	Origin of Replication
**pSEVA2319[g1]-ChnR**	1	pL0-ChnR-AI	pSEVA2319[g1]	1	BsaI	TGCC	GCAA	Km	pBBR1
**pSEVA2519[g2]-P_m-G**	1	pL0-P_m-AB<br>pL0-BCD2-BD<br>pL0-msfGFP-DG<br>pL0-rnpB_T1-GI	pSEVA2519[g2]	2	BsaI	GCAA	ACTA	Km	RSF1010
**pSEVA2519[g2]-P_{rhaBAD}-G**	1	pL0-P_{rhaBAD}-AB<br>pL0-BCD2-BD<br>pL0-msfGFP-DG<br>pL0-rnpB_T1-GI	pSEVA2519[g2]	2	BsaI	GCAA	ACTA	Km	RSF1010
**pSEVA2319EliR[g2]-P_{JExD}-G**	1	pL0-P_{JExD}-AB<br>pL0-BCD2-BD<br>pL0-msfGFP-DG<br>pL0-rnpB_T1-GI	pSEVA2319EliR[g2]	2	BsaI	GCAA	ACTA	Km	pBBR1
**pSEVA2319[g2]-P_b-G**	1	pL0-P_b-AB<br>pL0-BCD2-BD<br>pL0-msfGFP-DG<br>pL0-rnpB_T1-GI	pSEVA2319[g2]	2	BsaI	GCAA	ACTA	Km	pBBR1
**pSEVA2819[g3]-P_{BAD}-G**	1	pL0-P_{BAD}-AB<br>pL0-BCD2-BD<br>pL0-msfGFP-DG<br>pL0-rnpB_T1-GI	pSEVA2819[g3]	3	BsaI	ACTA	TTAC	Km	pMB1
**pTn7-19[gE]-P_m-G**	2	pSEVA2819[g1R]-XylS<br>pSEVA2519[g2]-P_m-G<br>pSEVA181-L_34<br>pSEVA181-L_45<br>pSEVA181-L_56<br>pL1F-Dummy_6	pTn7-19[gE]	E	BpiI	AGGT	TTCG	Gm/Km	R6K

Name	Level	Component Plasmid(s)	Backbone	Position	Restriction Enzyme†	5’ Fusion site	3’ Fusion site	Antibiotic Resistance	Origin of Replication
**pTn7-19[gE]-P$_{rhaBAD}$-G**	2	pSEVA2319[g1]-RhaSR<br>pSEVA2519[g2]-P$_{rhaBAD}$-G<br>pSEVA181-L_34<br>pSEVA181-L_45<br>pSEVA181-L_56<br>pL1F-Dummy_6	pTn7-19[gE]	E	BpiI	AGGT	TTCG	Gm/Km	R6K
**pTn7-19[gE]-P$_{JExD}$-G**	2	pSEVA2819[g1R]-EliR<br>pSEVA2319EliR[g2]-P$_{JExD}$-G<br>pSEVA181-L_34<br>pSEVA181-L_45<br>pSEVA181-L_56<br>pL1F-Dummy_6	pTn7-19[gE]	E	BpiI	AGGT	TTCG	Gm/Km	R6K
**pTn7-19[gE]-P$_b$-G**	2	pSEVA2319[g1]-ChnR<br>pSEVA2319[g2]-P$_b$-G<br>pSEVA181-L_34<br>pSEVA181-L_45<br>pSEVA181-L_56<br>pL1F-Dummy_6	pTn7-19[gE]	E	BpiI	AGGT	TTCG	Gm/Km	R6K
**pTn7-19[gE]-P$_{BAD}$-G**	2	pSEVA2319[g1]-P$_{J106}$-AraC<br>pSEVA181-L_23<br>pSEVA2819[g3]-P$_{BAD}$-G<br>pSEVA181-L_45<br>pSEVA181-L_56<br>pL1F-Dummy_6	pTn7-19[gE]	E	BpiI	AGGT	TTCG	Gm/Km	R6K

† Restriction enzyme used to make construct; Gm, gentamicin; Km, kanamycin; Ap: ampicillin

Table S8. **Evaluation of the synthetic promoter library in *B. bacteriovorus* HD100**

	Inoculum				Microplate experiment			
Strain	Activity (%) [a]	Log (FC events · mL^{-1}) [b]	Log (initial PFUs · mL^{-1}) [c]	Log (final FC events · mL^{-1}) [d]	OD_{600} curve		Fluorescence curve	
					r_{max} (10^{-2} h^{-1}) [e]	s (h) [f]	s (h) [g]	p_{max} (a.u.) [h]
**HD100**	N.D.*	9.33 ± 0.10	7.35 ± 0.04	9.23 ± 0.07	-4.10 ± 0.54	14.33 ± 1.68	N.D.*	N.D.*
**Bb08-ncTU**	N.D.*	9.31 ± 0.07	7.30 ± 0.05	9.22 ± 0.05	-3.18 ± 0.32	14.74 ± 0.96	N.D.*	N.D.*
**Bb08-P_0-R**	N.D.*	9.22 ± 0.04	7.35 ± 0.06	9.17 ± 0.10	-4.00 ± 0.45	14.04 ± 0.63	N.D.*	N.D.*
**Bb08-P_{J106}-R**	11.12 ± 0.51	9.28 ± 0.09	7.32 ± 0.02	9.18 ± 0.05	-3.82 ± 0.13	13.83 ± 0.84	14.91± 0.69	7.84 ± 0.32 · 10^4
**Bb08-P_{J116}-R**	39.58 ± 1.36	9.33 ± 0.04	7.33 ± 0.03	9.17 ± 0.11	-3.85 ± 0.13	14.86 ± 0.94	14.18 ± 0.60	4.85 ± 0.24 · 10^5
**Bb08-P_{BG37}-R**	75.21 ± 0.73	9.27 ± 0.08	7.33 ± 0.04	9.14 ± 0.05	-3.94 ± 0.20	14.80 ± 1.55	14.35 ± 0.97	9.13 ± 0.15 ·10^5
**Bb08-P_{J102}-R**	87.09 ± 0.34	9.29 ± 0.08	7.35 ± 0.04	9.16 ± 0.07	-3.83 ± 0.17	13.99 ± 1.10	14.52 ± 0.42	1.11 ± 0.07 · 10^6
**Bb08-P_{BG25}-R**	94.93 ± 1.09	9.31 ± 0.11	7.29 ± 0.06	9.16 ± 0.06	-3.95 ± 0.13	14.07 ± 0.62	14.83 ± 0.73	1.06 ± 0.08 · 10^6
**Bb08-P_{BG42}-R**	100	9.28 ± 0.08	7.26 ± 0.02	9.14 ± 0.03	-4.06 ± 0.15	13.63 ± 0.94	14.58 ± 0.70	1.17 ± 0.08 · 10^6

a Activity (%): Relative activity of each promoter, taking the highest expressing promoter as reference. Calculated with the median of the fluorescent population obtained analyzing by flow cytometry (FC) 24-hours predated co-cultures in flasks.
b Events · mL^{-1}: Total events measured by flow cytometry of a 24-h co-culture grown in flask that served as inoculum for the microplate experiment.
c Initial PFUs of the microplate experiment were measured following the double-layer method.
d Total events measured by flow cytometry of the predated co-cultures grown in a microplate reader.
e Maximal death rate (h^{-1}): Maximal rate of the OD_{600} absorbance decrease, i.e., the absorbance of the prey.
f Time in h when the decrease in the OD_{600} is maximal.
g Time in h when the increase of the fluorescence is maximal.
h Maximal fluorescence reached
e, **f**, **g,** and **h** were calculated using CuRveR. No statistical differences were found in **b**, **d**, **e** (ANOVA, P > 0.05), **f**, **g**, and **f** vs **g** (Brown-Forsythe ANOVA, P > 0.05).
***** N.D.: Not detected.

Table S9. Assessment of the impact of increasing concentrations of Crystal Violet (CV) on the Bb08-P_{JExD}-G strain in microplate experiment

Strain	[CV] (µM)	Log (initial PFUs · mL^{-1}) [a]	Log (final FC events · mL^{-1}) [b]	OD_{600} curve		Fluorescence curve	
				r (10^{-2} h^{-1}) [c]	s (h) [d]	s (h) [e]	p_{max} (10^6 a.u.) [f]
**HD100**	0	7.29 ± 0.02	9.17 ± 0.04	-3.84 ± 0.57	13.60 ± 0.14	N.D.*	N.D.*
**Bb08-P_{JExD}-G**	0	7.28 ± 0.04	9.21 ± 0.04	-3.98 ± 0.15	13.74 ± 1.68	12.88 ± 1.24	0.08 ± 0.01
	0.001		9.18 ± 0.04	-4.05 ± 0.25	12.99 ± 0.76	14.81 ± 1.66	0.52 ± 0.08
	0.005		9.18 ± 0.02	-4.07 ± 0.81	13.38 ± 0.70	13.73 ± 0.92	0.66 ± 0.12
	0.02		9.15 ± 0.01	-3.46 ± 0.22	14.35 ± 0.20	13.62 ± 0.21	9.33 ± 1.87
	0.1		9.15 ± 0.01	-3.80 ± 0.25	14.14 ± 1.01	14.62 ± 0.45	47.53 ± 4.74
	0.5		9.13 ± 0.02	-3.76 ± 0.30	14.03 ± 0.03	15.10 ±0.23	58.95 ± 3.94
	1		9.15 ± 0.03	-3.38 ± 0.95	14.51 ± 0.84	14.45 ± 0.34	56.78 ± 4.53

a Initial PFUs of the microplate experiment measured following the double-layer method.
b Total events measured by flow cytometry (FC) of the predated co-cultures grown in a microplate reader.
c Maximal death rate (h^{-1}): Maximal rate of the OD_{600} absorbance decrease, i.e., the absorbance of the prey.
d Time in h when the decrease in the OD_{600} is maximal.
e Time in h when the increase of the fluorescence is maximal.
f Maximal fluorescence reached
c, **d**, **e,** and **f** were calculated using CuRveR. No statistical differences were found in **a** (*t*-test, P > 0.05), **b** (ANOVA, P > 0.05), **c**, **d,** and **e** (Brown-Forsythe ANOVA, P > 0.05).
* N.D.: Not detected.

Supplementary Figures

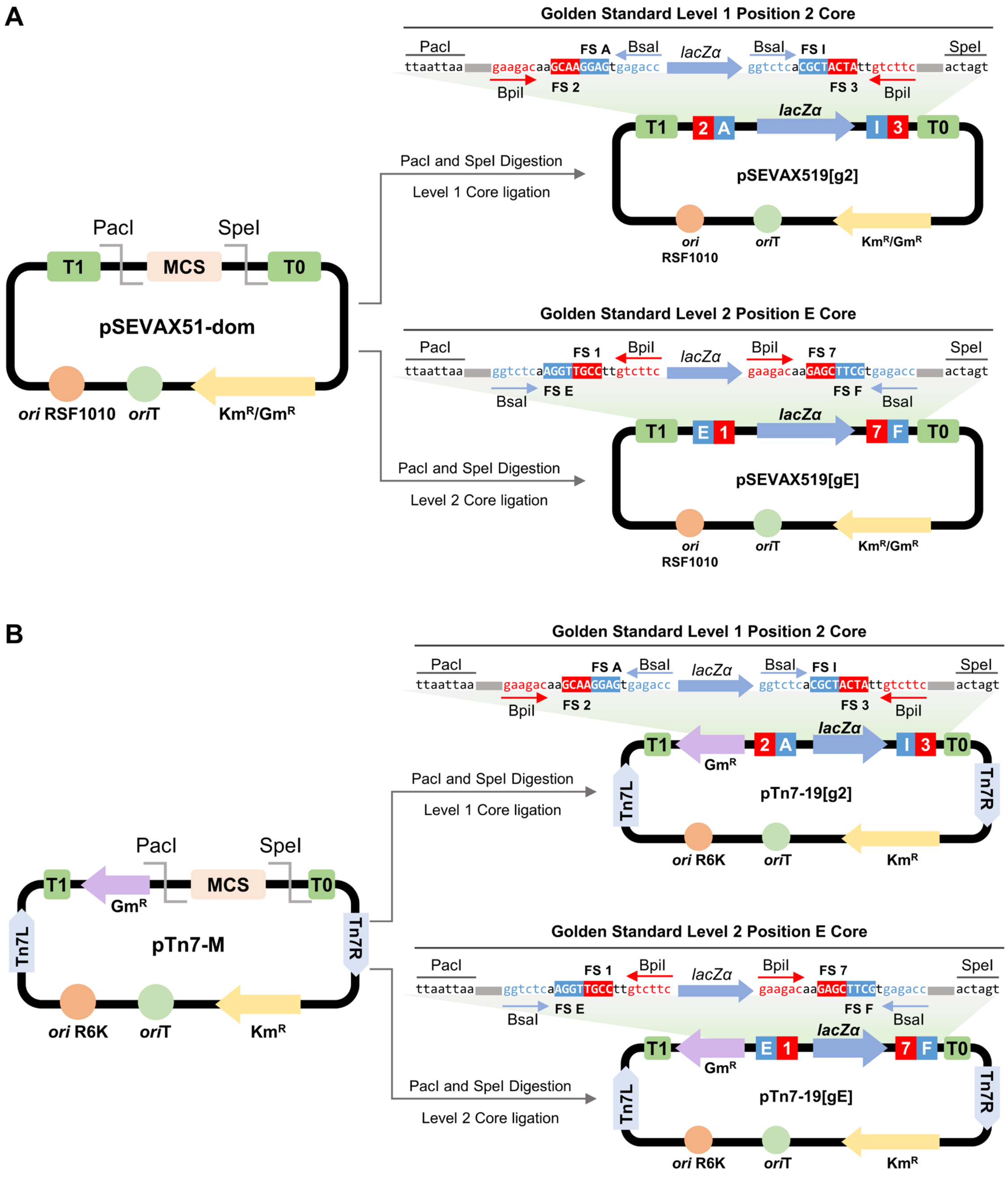

Figure S1. GS adapted plasmids. A) Plasmids bearing ori RSF1010 adapted for GS cloning system. The domesticated plasmids pSEVA251-dom and pSEVA651-dom, denoted by pSEVAX51-dom, were PacI/SpeI digested and further ligated with the GS Level 1 position 2 and Level 2 position E cores, yielding the plasmids pSEVAX519[g2] and pSEVAX519[gE], where X is 2 and 6, i.e., Km and Gm, respectively. In red are the fusion sites generated by BpiI, and in blue are the fusion sites generated by BsaI. **B) pTn7-M vector adapted for GS cloning system.** The pTn7-M plasmid was PacI/SpeI digested and further ligated with the GS Level 1 position 2 and Level 2 position E cores, yielding the plasmids pTn7-19[g2] and pTn7-19[gE]. In red are the fusion sites generated by BpiI, and in blue are the fusion sites generated by BsaI.

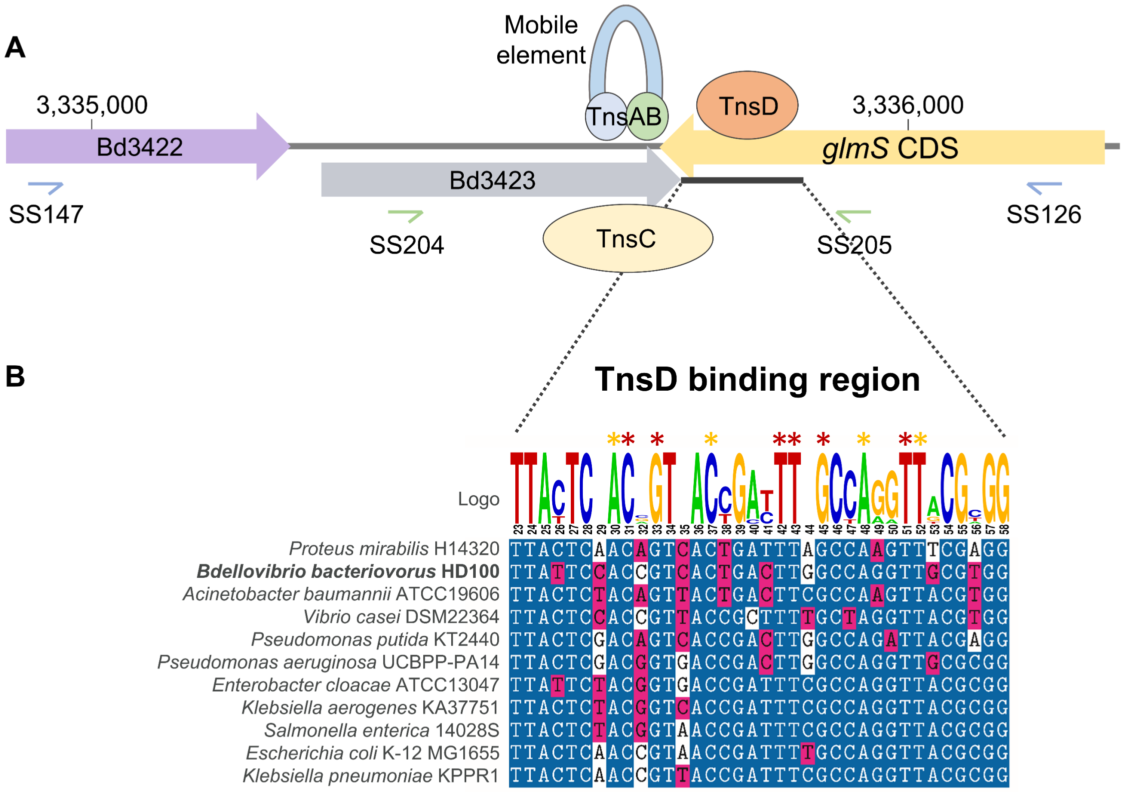

Figure S2. Analysis of the attTn7 site in *B. bacteriovorus* HD100. A) Localization of the attTn7 site in the genome of *B. bacteriovorus* HD100 and scheme of the transposase recognition site. The chromosomal integration mediated by the transposon Tn7 comprises four proteins TnsA, TnsB, TnsC, and TnsD. TnsD is the protein that binds to the 3' region of the glmS gene. TnsC is a protein that joins the transposase (TnsAB) and the TnsD protein (Peters, 2014). The machinery for the mobile element transposition can be supplied in a suicide plasmid (pTnS-1), avoiding the mobile element dispersion and off-target events (Choi et al., 2005). B) Multiple alignment of experimentally validated TnsD binding regions. The consensus logo marks critical nucleotides for TnsD binding with a red asterisk. Nucleotides whose mutation has a lower effect on TnsD binding are marked with a yellow asterisk (Mitra et al., 2010). Logo was generated with WebLogo (https://weblogo.berkeley.edu/logo.cgi), using default colors. Alignment was done with ClustalO 1.2 with the R package 'msa' (Bodenhofer et al., 2015).

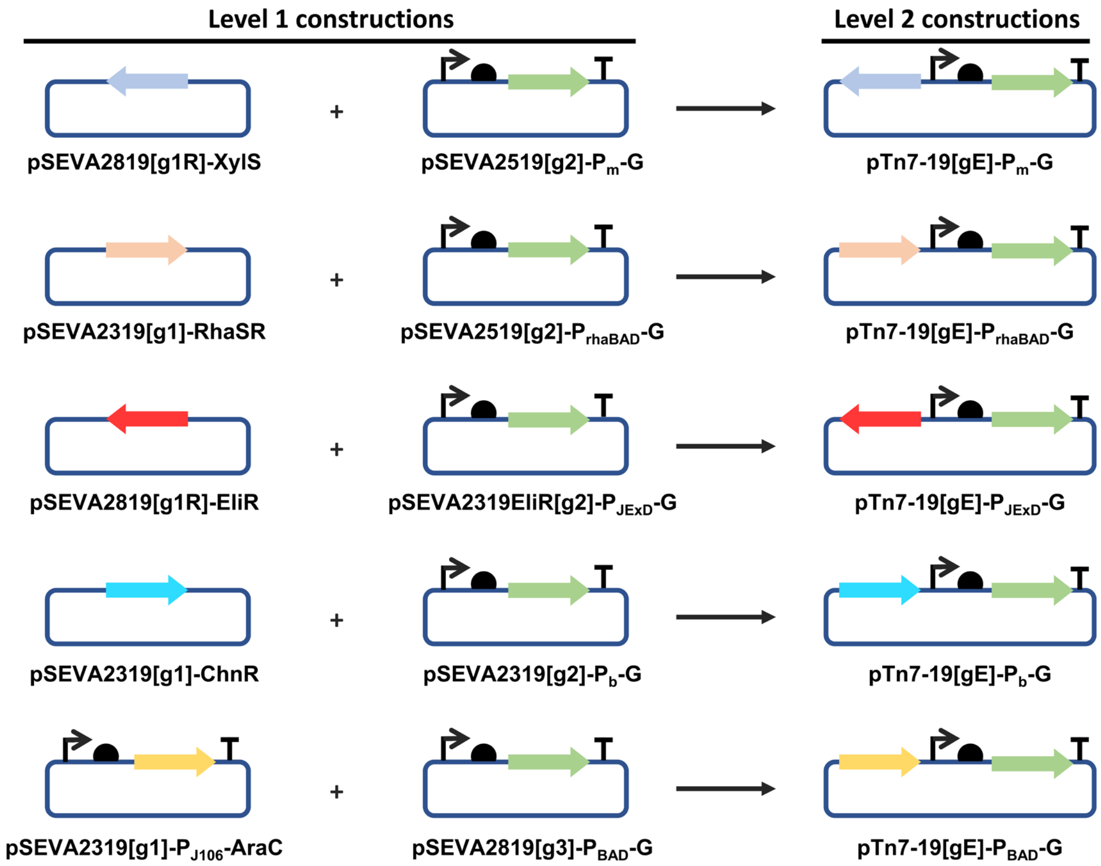

Figure S3. GS constructions made for the inducible expression of *msfGFP* in *B. bacteriovorus* HD100. The *msfGFP* CDS was assembled with the P_m, P_{rhaBAD}, P_{JExD}, P_b, and P_{BAD} promoters, keeping constant the other parts, i.e., the translational coupler BCD2 and the rnpB_T1 terminator, yielding five Level 1 constructions. These Level 1 constructions were mixed with the Level 1 plasmids carrying the regulator of each inducible system and the Level 2 destination vector, pTn7-19[g2], yielding five Level 2 plasmids with the MsfGFP constructions in the Tn7 mobile element.

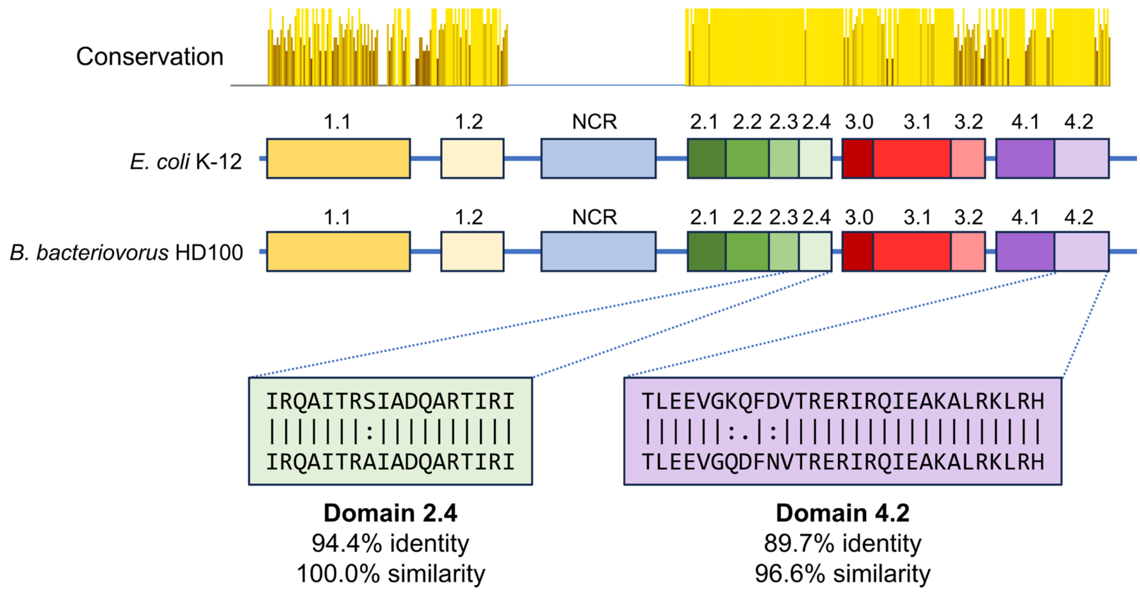

Figure S4. Comparison of the RpoD σ factors of *E. coli* K-12 and *B. bacteriovorus* HD100. Domains of each RpoD are schematically represented, with their amino acid conservation represented above (in yellow bars, with increased darkness indicating lower conservation). The sequence of the subdomains involved in the recognition of -35 and -10 promoter's boxes (the subdomain 4.2 and 2.4, respectively) are compared below.

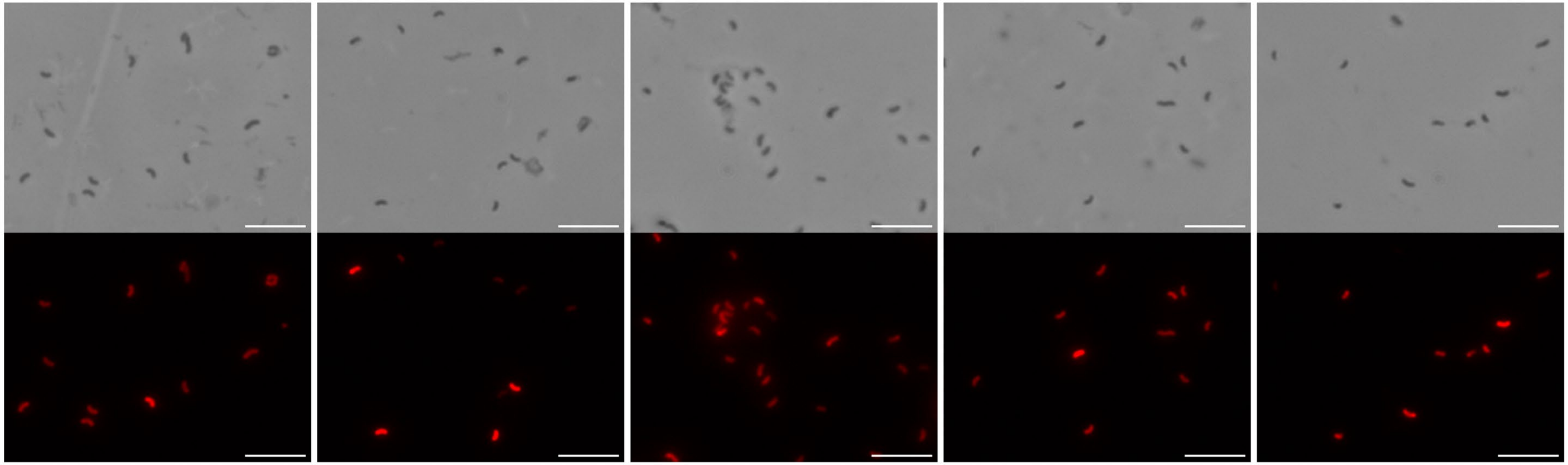

P_{J102} P_{J116} P_{BG25} P_{BG37} P_{BG42}

Figure S5. Phase-contrast and fluorescence microscopy images of mRFP1 harboring plasmids *B. bacteriovorus* HD100 strains. Pictures of 24-hours of co-cultures in Hepes+ supplemented with 5 µg · mL^{-1} of Gm and *E. coli* BL21(DE3) Gm^R as prey.

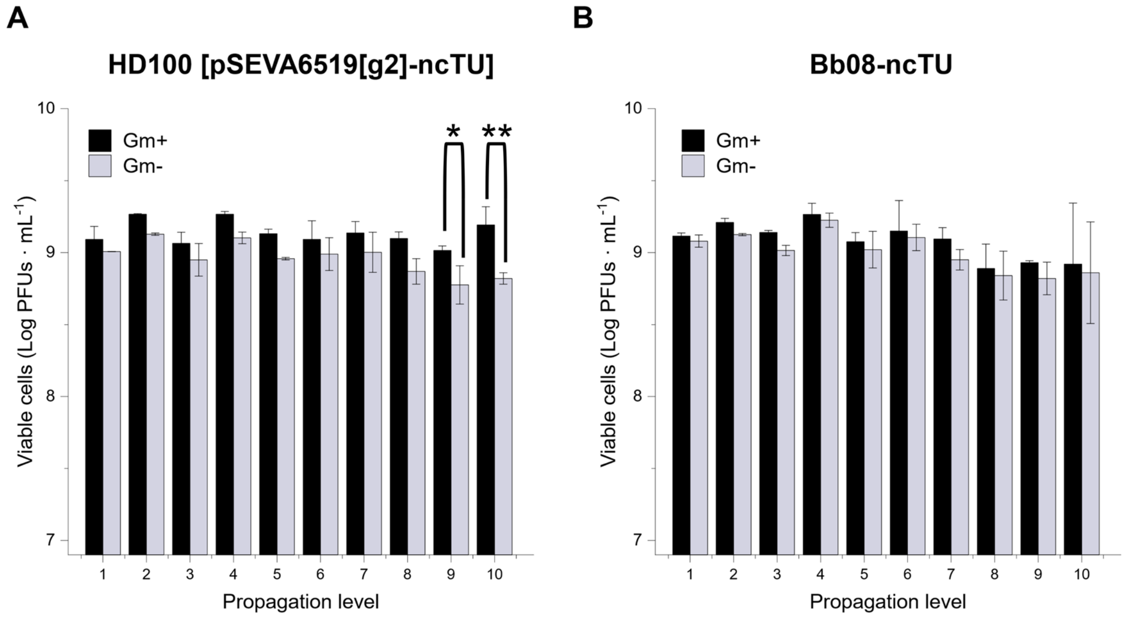

Figure S6. Stability of plasmid vs Tn7 integration in *B. bacteriovorus* HD100*.* *B. bacteriovorus* Gm^R viable cells in successive co-cultures with and without antibiotic pressure. **A)** Data obtained from the strain carrying the conferring resistance plasmid, pSEVA6519[g2]-ncTU. **B)** Data obtained from the strain with the Gm^R cassette in the chromosome, Bb08-ncTU. The strains were grown in *E. coli* BL21(DE3) Gm^R suspensions at OD_{600} 1 in $Hepes^+$. Error bars represent the SD of two biological replicates. Two-way ANOVA with Bonferroni post-test was applied to compare results within each propagation level (* means $P < 0.1$ and ** $P < 0.05$).

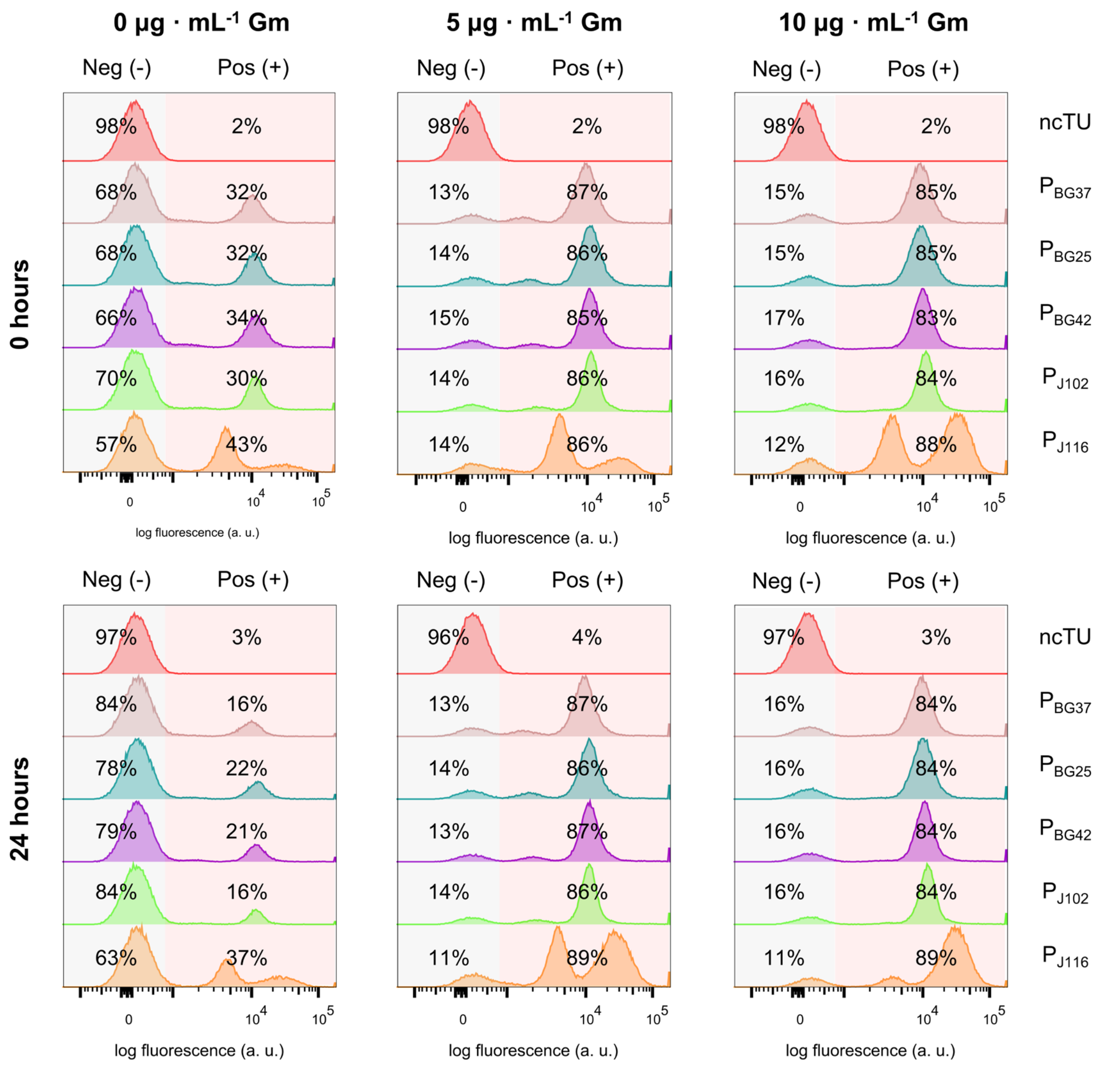

Figure S7. Evolution of fluorescent populations of plasmids bearing *B. bacteriovorus* HD100 strains. Two successive pre-cultures were grown supplementing with 10 µg · mL^{-1} Gm using *E. coli* BL21(DE3) Gm^R as prey at OD_{600} 1 in Hepes$^+$. The second pre-culture was measured by flow cytometry (0 h) and was used to inoculate fresh prey supplementing with the indicated Gm concentrations. After 24 h, the predated co-cultures were analyzed again by flow cytometry (24 h). In the histograms are represented the percentage of positive (+) and negative (-) events over the total events. The name of the plasmids is represented by the promoter of each construction, the backbone for all the GS constructions is pSEVA6519[g2].

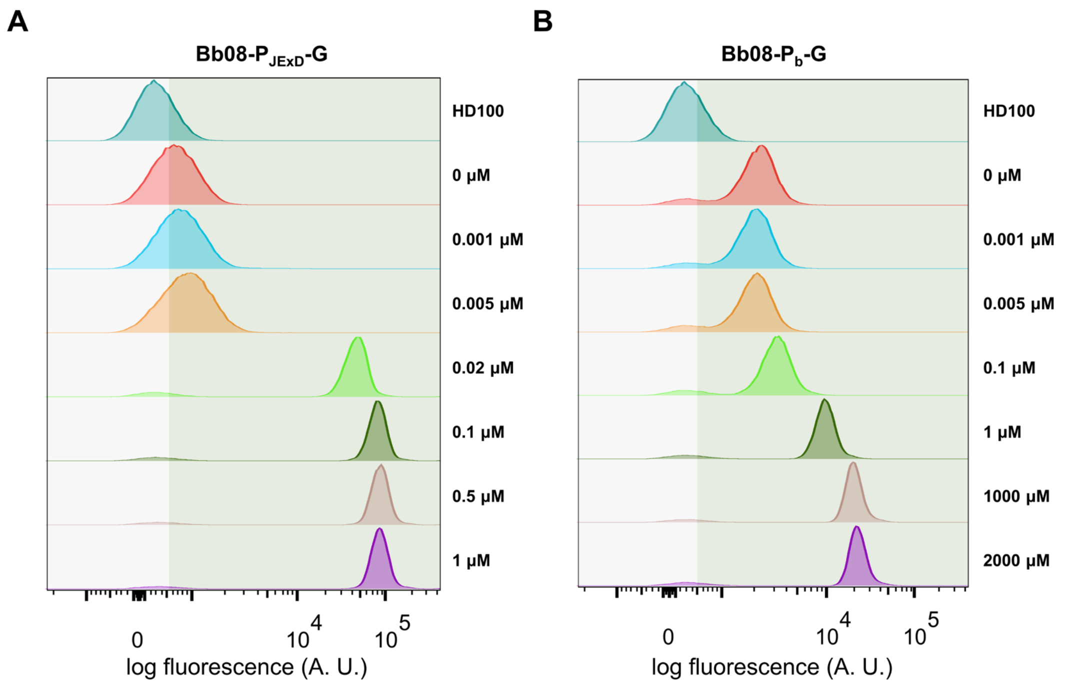

Figure S8. Single-cell fluorescence analysis of *B. bacteriovorus* strains with inducible promoters chromosomal integrated. The strains were grown in *E. coli* BL21(DE3) Gm^R suspensions at OD_{600} 1, in $Hepes^+$ supplemented with the indicated concentrations of the respective inducers. At 24 h, the predated co-cultures were analyzed by flow cytometry. *B. bacteriovorus* HD100 was used as the negative control. **A)** Stacked histogram corresponding to the Bb-P_{JExD}-G strain. **B)** Stacked histogram corresponding to the Bb-P_b-G strain.
